# Synthesis of atom-precise supported metal clusters *via* solid-phase peptide synthesis†

**DOI:** 10.1039/d4sc04400b

**Published:** 2024-08-16

**Authors:** Takane Imaoka, Nanami Antoku, Yusuke Narita, Kazuki Nishiyama, Kenji Takada, Shogo Saito, Masayoshi Tanaka, Mina Okochi, Miftakhul Huda, Makoto Tanabe, Wang-Jae Chun, Kimihisa Yamamoto

**Affiliations:** a Laboratory for Chemistry and Life Science, Tokyo Institute of Technology Yokohama 226-8503 Japan timaoka@res.titech.ac.jp yamamoto@res.titech.ac.jp; b Department of Chemical Science and Engineering, Tokyo Institute of Technology Tokyo 152-8552 Japan; c Graduate School of Arts and Sciences, International Christian University Mitaka Tokyo 181-8585 Japan

## Abstract

While the utility of supported metal and alloy clusters as catalytic materials is widely recognized, their precise synthesis remains a challenge. Here, we demonstrate the precise synthesis of these clusters *via* metallopeptides. This technique is characterized by its ability to be automated using Merrifield's solid-phase peptide synthesis (SPPS). Metallopeptides with iron and platinum complexes in their side chains have been prepared using this SPPS. These metallopeptides were successfully transformed into the corresponding supported metal clusters by heating in a hydrogen atmosphere.

## Abstract 2

A new approach enables the automated synthesis of metal and alloy clusters, using metallopeptides as precursors. This method employs Merrifield's solid-phase peptide synthesis, which are then transformed into metal clusters.

## Graphical abstract

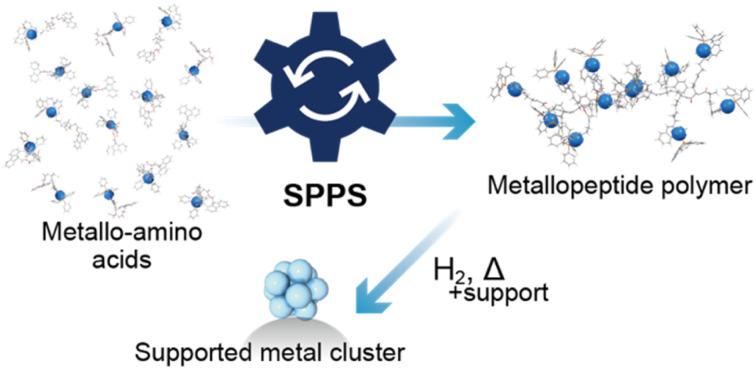

## Introduction

Subnanometer metal particles, including what are commonly referred to as metal clusters, have garnered significant attention due to their distinct properties, which set them apart from larger metal nanoparticles. These unique attributes contribute not only to the development of functional materials for applications such as catalysis^1–6^ and luminescence^7^ but also to the unexpected emergence of super-atomic properties.^8–11^ What is particularly intriguing is the absence of phase separation in these clusters, which allows for the flexible creation of alloy formations with a wide variety of elements.^12–15^ Given the countless possibilities for subnanometer alloys, conducting high-throughput searches for materials within this nearly limitless spectrum of alloyed clusters is an essential prerequisite for future research endeavors.

There are numerous reports on stable series of clusters such as polyoxometalates,^16^ multi-metallic clusters based on the composition of Zintl anions,^17^ and ligand-protected metal clusters.^18,19^ These clusters are often exceptionally stable and are discovered serendipitously or based on empirical conjecture. In contrast are the meta-stable clusters supported on solid substrates. These clusters are also known as transient active species during the operation of single-atom catalysts and metal-supported catalysts,^20^ and many remain unexplored as they do not always adopt the most stable structures.^21–23^

The difficulty of studying such meta-stable clusters is attributed to significant constraints in their synthesis methods. Nonetheless, such clusters potentially exhibit outstanding catalytic activity and cannot be overlooked.^24–29^ Because the metastability of such clusters makes their isolation challenging, they are typically synthesized in the gas phase, and are then separated in flight using a quadrupole mass filter and gently deposited on substrates.^28,30–32^ This method limits throughput, posing challenges when attempting catalytic reactions on a practical scale. Furthermore, in the case of alloy clusters, the complexity of the products complicates separation.

**Fig. 1:**
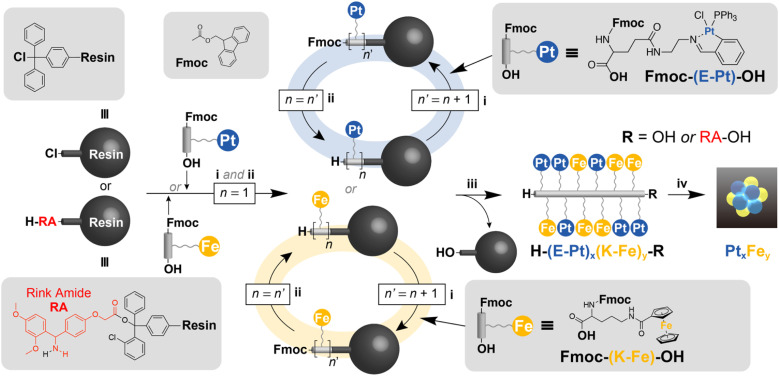
Synthesis of platinum- and iron-containing metallopeptides by Merrifield's solid-phase peptide synthesis (SPPS) for atom-precise cluster synthesis. (i) Elongation of the peptide chain and capping of the residual amino groups, (ii) deprotection of the Fmoc group: 20% piperidine/DMF (2 mL), 15 min, (iii) cleavage of the peptide chain: 1% TFA solution, (iv) deposition on the support and calcination: Ketjenblack, H_2_/N_2_ (3%) flow, 250 °C, 3 h.

Recent advancements in the precision synthesis of small nanoparticles^33–36^ and clusters^1,37,38^ using organic polymers and self-assembling molecules as templates have allowed access to these materials. The remaining issue is the necessity for a complex, multistep organic synthesis and repeated purification processes to custom-tailor specific template molecules for each desired cluster product. Since the most suitable template must be selected according to each desired cluster, screening through automated synthesis in a single protocol is not yet feasible. This study aims to demonstrate the feasibility of achieving template-free cluster synthesis with atomic-level precision using Merrifield's solid-phase peptide synthesis (SPPS)^39^ towards a universal protocol. The concept of the entire synthesis process is to directly convert automatically synthesized metallopeptides into the desired clusters, as shown in Fig. 1.

## Results and discussion

### Preliminary study and design of metallated amino acids

The design of metal-containing peptides involves the careful selection of specific amino acids with ligands that act as scaffolds for metal complexes. In basic molecular design, it is crucial to first consider the timing of metal complex binding, and then identify the segment of the amino acid to which the metal complex will be attached. We introduced the complexes to the amino acids before starting the peptide chain elongation process, rather than adding the complexes after the peptide chain was fully extended. Although there are several examples^40–42^ of introducing metal complexes after the elongation process, we refrained from adopting this approach because it is difficult to introduce multiple types of metal ions in precise numbers to multiple free ligands and form complexes with exact compositions.

We used “metallated α-amino acids” as the basic building blocks, with α-amino acids forming the core structure and metal complexes attached to their side chains. After preliminary investigation, we judiciously selected two types of metallated amino acids (Fe, Pt) (Fig. 1) based on stability and accessibility considerations. To complete the entire process as shown in Fig. 1, the side-chain metal complex must have the chemical stability not to react during the repeated condensation (i and ii) and the cleavage (iii). On the other hand, during the thermal decomposition to produce the cluster product (iv), the ligand part must be cleanly removed without leaving any residue. From the perspective of chemical stability, polypyridine and macrocyclic complexes (such as porphyrins) are good options. However, in the decomposition process (iv), these complexes produced graphitic ash as the side product in addition to the metallic component. In this study, we employed iron cyclopentadienyl complexes and platinum Schiff base complexes as ligands that possess both chemical stability and ease of thermal decomposition.

### Synthesis of Fe metallo-peptides [H-(K-Fe)_12_-RA-OH]

To the best of our knowledge, the long-chain metallo-peptide targeting 12 residues of metalized amino acids has never been synthesized before. Therefore, we attempted to optimize the solid-phase peptide synthesis method for metallated amino acids and to synthesize the long-chain metallo-peptide. Fig. 1 illustrates the procedure for the synthesis of iron-containing metallopeptides involving the attachment of a ferrocene group to a C-terminally protected lysine followed by the subsequent deprotection of the monomers (Fmoc-(K-Fe)-OH). Further details of the synthesis can be found in the ESI.† It is noteworthy that Fmoc-(K-Fe)-OH showed remarkable stability when dissolved in a 1% trifluoroacetic acid (TFA)/dichloromethane (DCM) solution. However, it was susceptible to damage when exposed to a 95% TFA solution. Therefore, in this study, we used acid-sensitive resins that could be cleaved using a 1% TFA solution for SPPS.

In order to efficiently obtain the desired metallopeptide, it is crucial to increase the efficiency of peptide elongation. In particular, the choice of condensing agent plays a key role in determining the yield and purity of the target product, as it directly affects the reaction efficiency and side reactions, primarily racemization. Our preliminary investigations have shown that 1-[bis(dimethylamino)methylene]-1*H*-1,2,3-triazolo[4,5-*b*]pyridinium 3-oxide hexafluorophosphate (HATU) is the most suitable coupling agent for the synthesis of the metallopeptides in this study. 1-[(1-(Cyano-2-ethoxy-2-oxoethylideneaminooxy) dimethylaminomorpholino)] uronium hexafluorophosphate (COMU) was also effective as a coupling agent, but its stability proved to be less favorable as the number of condensation cycles increased. Therefore, we decided to use HATU as the coupling agent along with 3*H*-[1,2,3]triazolo[4,5-*b*]pyridin-3-ol (HOAt) as the co-coupling agent and *N*,*N*-diisopropylethylamine (DIEA) as the base agent to achieve optimal results.

We performed an elongation study in which the peptide chain was extended up to 12 residues on a 0.027 mmol scale using Fmoc-(K-Fe)-OH as the monomer. We used Rink amide-Trt(2-Cl) resin packed into a column that was first swollen with DCM. Fmoc-(K-Fe)-OH (3 equiv.) was then added along with HATU (3.0 equiv.), HOAt (2.9 equiv.), and DIEA (6.0 equiv.). The mixture was then subjected to microwave irradiation at 75 °C for 5 minutes. A TNBS (2,4,6-trinitrobenzenesulfonic acid) assay performed after peptide condensation confirmed the absence of residual amino groups, indicating quantitative condensation. After filtration of the reaction solution, any remaining active sites (–NH_2_) were deactivated by treatment with an excess of acetic anhydride to prevent further extension (capping). The Fmoc protecting group at the end of the growing peptide chain was then removed using a 20% piperidine/dimethylformamide (DMF) solution. For a detailed sequence of operations and conditions, refer to the ESI.†

**Fig. 2:**
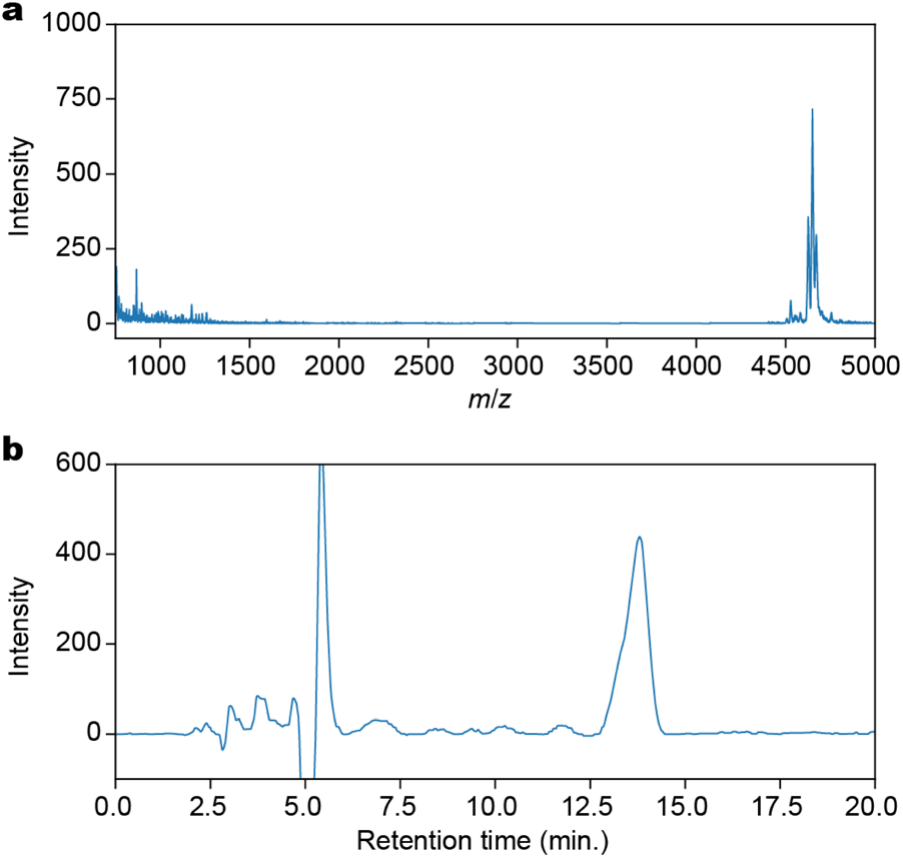
Characterization of H-(K-Fe)_12_-OH synthesized by the SPPS method. (a) A MALDI-TOF-mass spectrum. (b) A reversed-phase analytical HPLC chart. The solvent was 90% DMF/H_2_O.

The condensation, capping, and deprotection process was iterated 12 times to produce a 12-residue Fe peptide attached to the resin. A cleavage cocktail consisting of TFA : triisopropylsilane (TIPS) : 2,2,2-trifluoroethanol (TFE) : DCM in a ratio of 1 : 2.5 : 30 : 66.5 was added to the resin. This mixture was filtered and the process was repeated until the filtrate became colorless. To precipitate the peptides, *tert*-butylethylether was added to the filtrate. The crude product was obtained by centrifugation at 3000*g* at 0 °C for 20 minutes, followed by decanting. The crude product was purified by preparative reverse-phase HPLC, yielding 8.9 mg (7.1%) of the desired product. The purified product, H-(K-Fe)_12_-RA-OH (RA: Rink Amide), was confirmed by Matrix Assisted Laser Desorption/Ionization Time-Of-Flight (MALDI-TOF)-mass spectrometry (Fig. 2a) with a purity of 94% as determined by HPLC (Fig. 2b).

### Synthesis of Pt metallopeptides [H-(E-Pt)_12_-OH]

SPPS was also used to prepare Pt metallopeptides. Although the design of is similar to that reported in the literature,^43,44^ we chose to use an amide linkage instead of an ester linkage to link the main chain to the side chain. This modification was made to increase the stability against acidic conditions. To initiate the synthesis, the monomer (Fmoc-(E-Pt)-OH) was attached to an activated resin. Subsequently, the similar condensation–deprotection procedures used for Fmoc-(K-Fe)-OH were repeated up to five times to produce a 6-residue metallopeptide with an NH_2_ group at the N-terminal end. The total number of cycles corresponds to the number of residues because the TNBS assay confirmed the completion of the second and subsequent condensations (>99.5%). After treatment with CH_2_Cl_2_ : TFA : TIPS in a ratio of 96.5 : 1.0 : 2.5 (v/v), the eluate was collected and then precipitated with cold diethyl ether. This procedure gave the product H-(E-Pt)*_n_*-OH (*n* = 1, 2, 3, 4, 6) after filtration.

Analytical HPLC chromatograms of the products H-(E-Pt)*_n_*-OH (*n* = 1, 2, 3, 4, 6) prior to purification are shown in Fig. S1.† Notably, the elution time in reversed-phase HPLC increased as the number of residues (*n*) increased. Importantly, there was no observable peptide contamination across different *n* values, suggesting that the condensation–deprotection process proceeded quantitatively as indicated by the TNBS assay. Upon completion of the purification process by preparative HPLC, we obtained 5.3 mg of a 6-residue peptide. The formation of metallopeptides with residue numbers ranging from 1 to 6 was confirmed by MALDI-TOF-MS analysis. The visualization of platinum atoms by Cs-corrected annular-dark-field scanning transmission electron microscopy (ADF-STEM) together with the simulation allowed us to unravel the conformational structures of H-(E-Pt)_12_-OH, revealing features such as entanglement and self-folding (Fig. S2†).

While it is true that the efficiency of the reaction decreased with increasing peptide chain length, even the final step of 12-residue metallopeptide formation was achieved with almost quantitative yield (∼98%). The final resin-bound product was cleaved using a 1% TFA solution, followed by recovery and isolation by preparative HPLC (Fig. S3†). MALDI-TOF-MS analysis of the 12-residue metallopeptide revealed a peak corresponding to the molecular weight of H-(E-Pt)_12_-OH, accompanied by fragment peaks (Fig. S4†). These fragments exhibited regular intervals similar to those observed for the pure H-(E-Pt)4–6-OH (Fig. S2†). The consistency of these intervals with the molecular weight of PPh_3_ suggests that these fragments are not the result of chemical decomposition in solution, but rather due to fragmentations occurring during the ionization process.

**Fig. 3:**
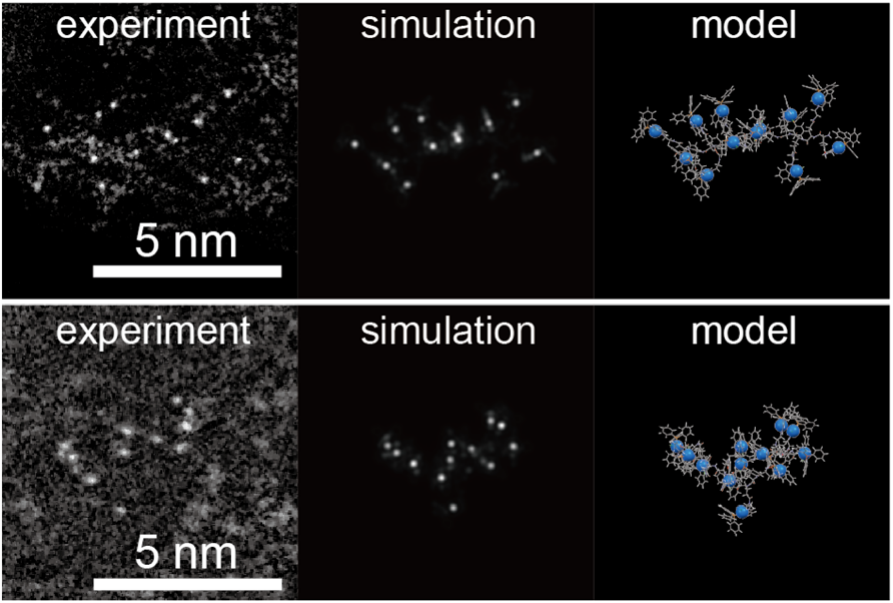
ADF-STEM images of H-(E-Pt)_12_-OH supported on Ketjenblack. The corresponding simulations and the projected molecular models are shown. A lower magnification image is shown in Fig. S5.†

Fig. 3 and S5† show images of H-(E-Pt)_12_-OH adsorbed on a carbon support (Ketjenblack). Numerous metallopeptides, each consisting of 12 bright spots, were individually observed on the support. In certain assemblies, platinum atoms were dispersed within a range of about 2 nm. Based on conformational searches using STEM simulations, this dispersion corresponds to the peptide chain extending outward, as shown in Fig. 3. In addition, we observed images of metals assembled in a more confined region. This likely corresponds to the peptide chains adopting different conformations, with the hydrophobic metal complexes aggregating together.

### Preparation of Pt clusters

**Fig. 4:**
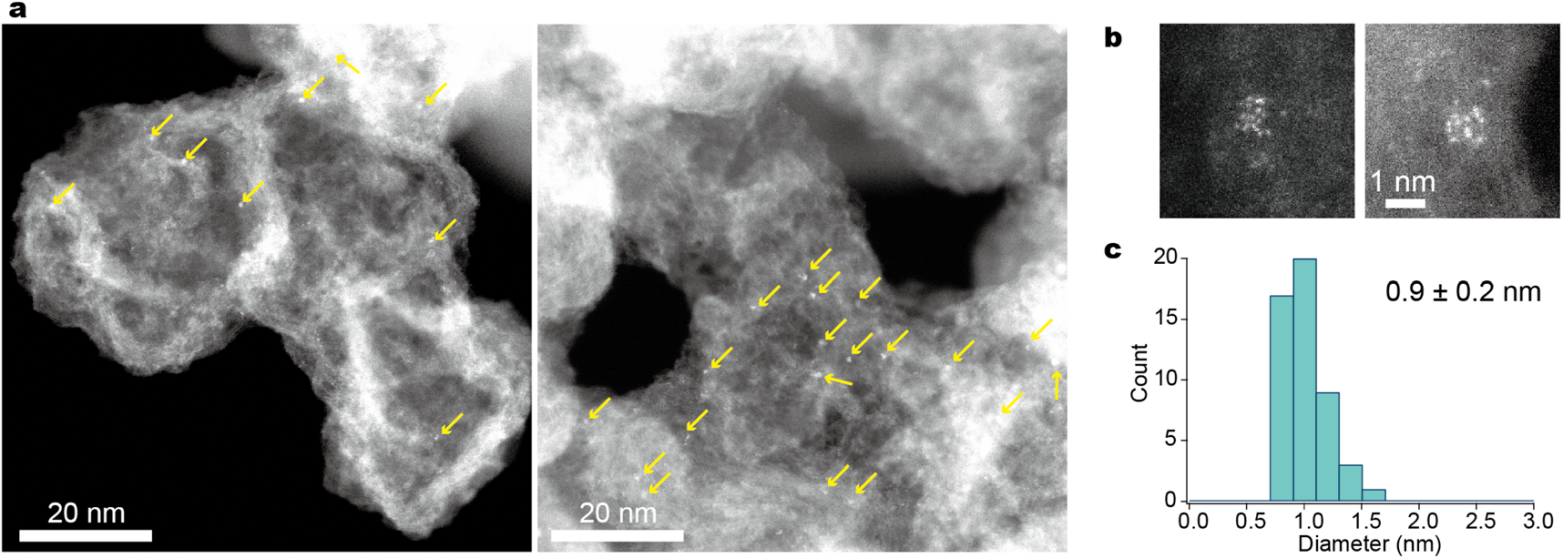
ADF-STEM images of Pt_12_/KB. (a) Wide-view images displaying the dispersion of clusters on the support. The arrows point to where the particles are located. It should be noted that many particles at the defocused area are unclear or invisible due to the narrow depth of field of Cs-corrected STEM (see Fig. S7†). (b) High-magnification images displaying each particle for atom counting. (c) A particle-diameter histogram from the low-magnification images.

As shown in Fig. 3, atomic resolution ADF-STEM images visualized H-(E-Pt)_12_-OH present without significant intermolecular entanglement when supported on Ketjenblack. The presence of the support material (Ketjenblack) played a crucial role in stabilizing these small metal clusters, as previously reported.^45^ The successful transformation of the metallopeptides into their corresponding supported metal clusters was achieved as follows. To obtain the desired metal clusters, the calcination of H-(E-Pt)_12_-OH on Ketjenblack was carried out at 250 °C for three hours under a hydrogen/nitrogen (3%) gas flow. This process efficiently yielded mainly zero-valent Pt metal clusters. This calcination condition is based on the results of evolved gas thermography and X-ray photoelectron spectroscopy (XPS) using H-(E-Pt)_4_-OH. The platinum peptide complexes initiated the reaction with H_2_ at 200 °C (Fig. S6†) and finally yielded zerovalent metal at 250 °C (Fig. S7a and b†). Furthermore, the disappearance of elements such as nitrogen, chlorine and phosphorus indicated the complete elimination of the peptide and ligand structures (Fig. S7c†). The ADF-STEM image confirmed the formation of metal clusters, which exhibited denser metal aggregates compared to the precursor complexes (Fig. 4 and S8†).

The decomposition of the Pt complex part starts at about 200 °C (Fig. S6b†), while the peptide backbone decomposes simultaneously, with a sharp gas desorption peak observed between 210 °C and 220 °C. Thus, in the structure of the metallopeptide proposed here, the complex part decomposes thermally, allowing the nearby free metal atoms to aggregate and form clusters. Subsequently, the main chain structure of the remaining peptide breaks down and is removed. This is believed to be the reason why the clusters could be synthesized with precision. In fact, attempts to synthesize clusters using complexes with polypridines^46^ or macrocyclic ligands,^47^ which have excessively high thermal stability, have not been successful. This failure is likely due to the main chain decomposing first, causing the complexes to scatter irregularly on the support.

The size distribution of the observed clusters was found to be 0.9 ± 0.2 nm (Fig. 4c), a size range comparable to that of clusters synthesized using tiara-like complexes^37^ and dendrimers.^24,48^ An atomic resolution video of a single Pt_12_ particle showing unique fluidity (Movie S1†) is consistent with the previous reports.^49,50^ Since STEM observations only provide localized information from a limited field of view, X-ray absorption fine structure (XAFS) was also measured at the Pt-L_3_ edge to complement the data (Fig. S9†). The X-ray absorption near-edge structure (XANES) and extended X-ray absorption fine structure (EXAFS) results are very similar to those of Pt_12_ obtained by the previous dendrimer-based method. This consistency rules out the possibility of single atoms not appearing in the STEM images or the presence of very large Pt particles out of the field of view. The presence of a Pt–Pt shell was further supported by curve-fitting analysis of EXAFS, as detailed in the ESI. †

Previous studies of tiara-like complexes^37^ have shown that the number of constituent atoms in the resulting clusters remains constant when the concentration is sufficiently low. In the current study, it was similarly observed that particle aggregation could be effectively suppressed when the amount of supported metallopeptide was kept below 2 wt% of the carbon support (with a Pt content of 0.5 wt%). While a small number of larger particles, each containing 24 Pt atoms, were also observed, atomic counting revealed that the majority consisted of the 12-atom clusters supported on Ketjenblack (Pt_12_/KB). In principle, the synthesis of smaller particles is possible, but achieving high purity in this context requires further refinements. This is due to the fact that when particle size is reduced while maintaining the loading mass fraction, the average distance between particles decreases, making them more prone to aggregation. A potential strategy to counteract this aggregation is the use of porous materials such as covalent organic frameworks (COFs)/metal organic frameworks (MOFs) and zeolites as support materials.^51,52^

Platinum clusters have exhibited outstanding catalytic performance, particularly in oxygen activation reactions like the oxygen reduction reaction (ORR)^25^ and aerobic oxidation reactions.^27^ In this study, the oxidation of toluene was selected as a benchmark reaction due to the efficient catalytic activity of subnanometer platinum in this process, as well as the well-established reproducibility and reusability of the catalyst.^27^

**Fig. 5:**
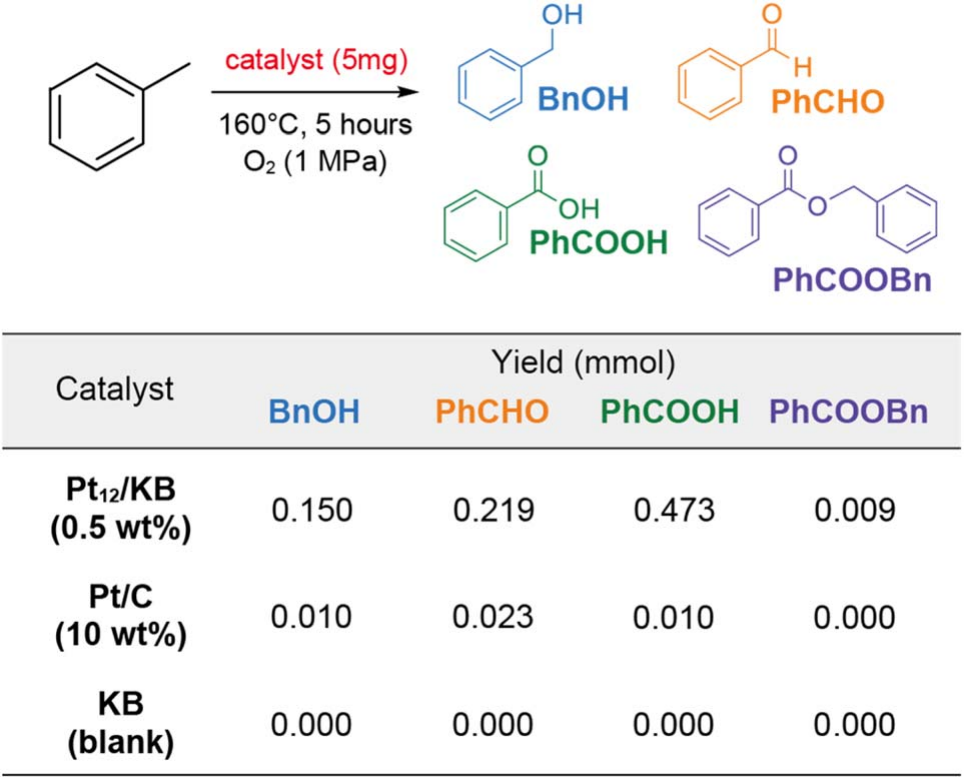
Catalytic oxidation of toluene by Pt catalysts. Reaction scheme and yields of products with different catalysts. Pt_12_/KB is the catalyst synthesized from the 12-residue metallopeptide. Pt/C is commercially available Pt supported on carbon. KB is the blank Ketjenblack which was used as the support in Pt_12_/KB.

In the experiment, 5 mg of Pt_12_/KB (0.47 wt%) was added to 2 mL of toluene and reacted at 160 °C under 1 MPa of oxygen for 5 hours without a solvent. The primary products, as determined by ^1^H NMR, included benzaldehyde, benzylalcohol, and benzoic acid (Fig. 5 and Table S1†). Additionally, benzylbenzoate was detected as a minor product. The total yield of products achieved with Pt_12_/KB was 0.85. In contrast, when an equivalent amount of Pt/C (commercial Pt on carbon, 10 wt%) was used, the product yield was substantially lower. The possibility of product formation *via* carbon-mediated auto-oxidation was ruled out, as no products were observed in the control experiment using platinum-free Ketjenblack (KB) as the catalyst. The nominal turn-over frequency (TOF) of Pt_12_/KB for each platinum atom was calculated to be 1330 times in 5 hours, confirming its activity was consistent with the TOF (∼1000) in a previous report.^27^ This result indicates that supported clusters synthesized using metallopeptides as precursors exhibit full catalytic activity without surface poisoning from unburned organic debris.

### Preparation of Pt–Fe bimetallic clusters

A precursor containing Pt and Fe atoms in equimolar amounts is necessary for the synthesis of useful magnetic materials such as L1_0_-FePt alloy. The FePt alloys are also known to be effective in oxygen reduction reactions (ORR) as catalysts.^53,54^ Although methods for the precise synthesis of FePt alloy nanoparticles (3–6 nm) using polymer metal complexes as templates have been reported,^55–57^ precise synthesis of clusters as small as 1 nm has not been achieved. Peptides containing two different metallic elements were alternately condensed and used as precursors to synthesize alloy clusters with precisely defined compositions. The metallopeptides used in this study contained both Fe and Pt elements. If the composition of the metallopeptides is well defined, the sequence order should not affect the composition of the resulting clusters. However, preliminary investigations have shown that condensation efficiency is enhanced when peptides of the same type are not extended sequentially. Therefore, to achieve an irregular sequence and improve condensation efficiency, we designed Fmoc-(E-Pt)-(K-Fe)-(E-Pt)_2_-(K-Fe)_2_-(E-Pt)-(K-Fe)_2_-(E-Pt)-(K-Fe)-(E-Pt)-RA (RA: Rink Amide) based on this idea.

The Fe–Pt metallopeptide was synthesized by SPPS on a 0.027 mmol scale, following a procedure similar to that used for the Fe and Pt metallopeptide. The resulting product appeared as a dark orange powder, which was subsequently dissolved in H_2_O/CH_3_CN = 50/50 and subjected to purification by reversed-phase HPLC. The MALDI-TOF-mass spectrum of the purified sample showed approximately two broad peaks (Fig. S10†). As explained previously, the broadening of these peaks can be attributed to the fragmentation of the phosphine ligands. The primary peak had an *m*/*z* significantly higher than 6150.4 (representing the peptide lacking a single Pt residue) and was in close proximity to 7080.6 (the target product). Based on these results, it can be concluded that the desired metallopeptide was successfully obtained. The collected product was 2.65 mg (1.3% yield).

For the synthesis of clusters, the Fe–Pt metallopeptide was used as a precursor. The metallopeptide was loaded on a carbon support (Ketjenblack or graphene powder) at 0.6 wt% metal content. It was sintered in a furnace at 300 °C for 2.5 h under a 3% H_2_ mixed N_2_ gas atmosphere to obtain clusters with the corresponding composition. The resulting carbon-supported clusters were directly observed by ADF-STEM and Energy Dispersive X-ray Spectroscopy (EDS) to identify their particle size and the elemental composition.

**Fig. 6:**
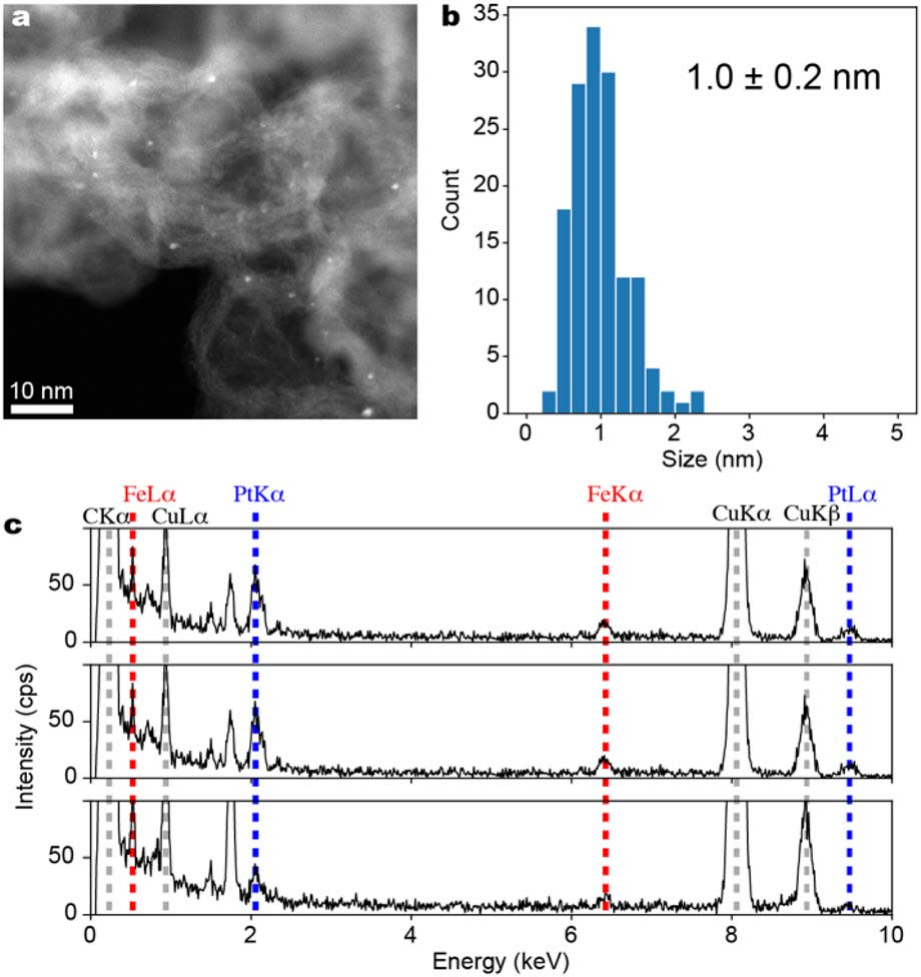
Characterization of Fe–Pt clusters (Fe_6_Pt_6_/KB). (a) ADF-STEM image with a wide field of view. (b) A histogram of particle diameters compiled from several ADF-STEM images, (c) Energy Dispersive X-ray Spectra (EDS) obtained from three different single particles. Spectra from all 11 fields of view are included in the ESI.†

The ADF-STEM images of the black powder obtained by calcination reduction of Fmoc-(E-Pt)-(K-Fe)-(E-Pt)_2_-(K-Fe)_2_-(E-Pt)-(K-Fe)_2_-(E-Pt)-(K-Fe)-(E-Pt)-RA supported on Ketjenblack are shown in Fig. 6a. Fe–Pt clusters are formed on Ketjenblack with a particle size of 1.0 ± 0.2 nm (Fig. 6b). EDS analysis targeting each individual cluster was conducted across 11 fields of view, confirming the coexistence of Pt and Fe in all fields. Although the quantitative accuracy of EDS was not satisfactory due to the very small particle size, it was revealed that the Pt/Fe ratio generally centered around 1 (Fig. 6c). The result support the idea that FePt alloy clusters with well-defined compositions are available from multi-element metallopeptides as the precursors.

## Conclusions

Merrifield's solid-phase peptide synthesis (SPPS) method has been used to successfully synthesize metallopeptides containing Fe and/or Pt complexes with up to 12 residues in an automated process. In addition, atom-precise clusters have been successfully synthesized from metallopeptides carrying platinum and iron complexes as the side chains. The successful synthesis of clusters (Pt_12_/KB and Fe_6_Pt_6_/KB) using metallopeptides as the precursors is mainly due to their pyrolysis process. One significant advantage of employing peptide sequences instead of traditional polynuclear metal complexes lies in high-throughput discovery. For instance, peptide arrays have proven effective in swiftly screening gold nanoparticles as templates to control size, shape, and optical properties.^58,59^ The concept presented in this study is expected to facilitate the automated synthesis of stable clusters and less stable clusters composed of various metal species with atomic precision. This advancement has the potential to expedite the search for catalysts, luminescent materials, and recording materials.

## Author contributions

N. A., Y. N., K. N., and S. S. were responsible for preparing the samples. T. I. and K. T. performed the Scanning Transmission Electron Microscopy (STEM) observations and processed the data. M. H. and M. T. conducted the catalyst benchmarking. T. I. and W. C. were in charge of the XAFS analysis. The experiments were conceptualized by T. I., M. T., M. O., and K. Y. The manuscript was co-written by T. I., M. T., M. O., and K. Y.

## Conflicts of interest

The authors declare no competing interests.

## Supplementary Material

SC-015-D4SC04400B-s001

SC-015-D4SC04400B-s002

## Data Availability

The data that support the findings of this study are available within the article and the ESI.†
