# Supplementary material for: Synthesis of atom-precise supported metal clusters via solid-phase peptide synthesis
Source: Chem Sci. 2024 Aug 16;15(36):14931–7. doi: 10.1039/d4sc04400b (PMC11376025; doi:10.1039/d4sc04400b)
Supplement: SC-015-D4SC04400B-s001 [file SC-015-D4SC04400B-s001.pdf]

Supplementary Information (SI) for Chemical Science.
This journal is © The Royal Society of Chemistry 2024

Electronic Supplementary Information for

Synthesis of Atom-Precise Supported Metal Clusters via Solid-Phase Peptide Synthesis

Takane Imaoka*[a,b], Nanami Antoku[a,b], Yusuke Narita[a,b], Kazuki Nishiyama[a,b], Kenji Takada[a], Shogo Saito[b], Masayoshi Tanaka[b], Mina Okochi[b], Miftakhul Huda[a], Makoto Tanabe[a], Wang-Jae Chun[c] and Kimihisa Yamamoto*[a,b]

a Dr. T. Imaoka, N. Antoku, Y. Narita, K. Nishiyama, Prof. Dr. K. Yamamoto
Laboratory for Chemistry and Life Science, Tokyo Institute of Technology, Yokohama 226-8503, Japan.
E-mail: timaoka@res.titech.ac.jp, yamamoto@res.titech.ac.jp

b Dr. T. Imaoka, N. Antoku, Y. Narita, K. Nishiyama, S. Saito, M. Tanaka, M. Okochi, Prof. Dr. K. Yamamoto
Department of Chemical Science and Engineering, Tokyo Institute of Technology, Tokyo 152-8552, Japan.

c Prof. Dr. W.-J. Chun
Graduate School of Arts and Sciences, International Christian University, Mitaka, Tokyo 181-8585, Japan.

This PDF file includes:

Supplementary Text
Figs. S1 to S10
Tables S1
References (1 to 2)

Other Supporting Materials for this manuscript include the following:

Movies S1

Abbreviations

SPPS: Solid-Phase Peptide Synthesis
TFA: Trifluoroacetic Acid
DCM: Dichloromethane
DMF: Dimethylformamide
TIPS: Triisopropylsilane
TFE: Trifluoroethanol
HATU: Hexafluorophosphate Azabenzotriazole Tetramethyl Uronium
HoAt: Hydroxy-7-azabenzotriazole
DIEA: Diisopropylethylamine
MALDI-TOF: Matrix-Assisted Laser Desorption/Ionization-Time of Flight
ADF-STEM: Annular Dark-Field Scanning Transmission Electron Microscopy
XPS: X-ray Photoelectron Spectroscopy
XAFS: X-ray Absorption Fine Structure
XANES: X-ray Absorption Near Edge Structure
EXAFS: Extended X-ray Absorption Fine Structure

1. Materials

N-(tert-Butoxycarbonyl)-1,2-diaminoethane, triphenylphosphine, 1-(3-Dimethylaminopropyl)-3-ethylcarbodiimide (EDCI), 4-dimethylamino pyridine (DMAP), Tetrakis(triphenylphosphine)palladium, Dimedone, Triisopropylsilane (TIPS) and *N, N*-Diisopropylethylamine were purchased from Tokyo Chemical Industry (TCI) CO., LTD. Benzaldehyde, Potassium tetrachloroplatinate(II) ($K_2[PtCl_4]$), trifluoroacetic acid were purchased from FUJIFILM Wako Pure Chemicals Co. Fmoc-*L*-glutamic acid 1-allyl ester was purchased from Watanabe Chemical Industry Inc. 1-[Bis(dimethylamino)methylene]-1*H*-1,2,3-triazolo[4,5-b]pyridinium 3-Oxide Hexafluorophosphate (HATU), and 1-Hydroxy-7-azabenzotriazole (HoAt) were purchased from Tokyo Chemical Industry (TCI) CO., LTD. Na_2CO_3, HCl, and the other solvents were purchased from Kanto Chemical Co., Inc. Ketjenblack (EC600JD: Primary particle size 34.0 nm, BET surface area 1270 $m^2\ g^{-1}$) was kindly provided by Lion Speciality Chemicals CO., LTD. as a sample. It is a conductive carbon black with a BET specific surface area of 1270 $m^2\ g^{-1}$ and a primary particle size of 34 nm. Pt/C (10wt%) catalyst was purchased from Sigma Aldrich.

2. Chemical analyses

The matrix-assisted laser desorption ionization time-of-flight mass spectra (MALDI-TOF-MS) were obtained using a spectrometer (Bruker, Ultraflex: Positive ion mode). The ^1H and ^{13}C nuclear magnetic resonance (NMR) measurements were recorded by an FT-NMR spectrometer (Bruker, Avance III 400) operating at 400 MHz (^1H) or 100 MHz (^{13}C). The ^1H NMR chemical shifts were referenced to the signal of tetramethoxysilane (0 ppm) as the internal standard. The ^{13}C NMR chemical shifts were referenced to the peak of $CDCl_3$ as the solvent (77.0 ppm). The X-ray photoelectron spectroscopy (XPS) measurements were carried out using a spectrometer (Ulvac-Phi, ESCA1700R) with Mg Kα radiation.

The X-ray absorption fine structure (XAFS) was measured in the transmission mode at the BL12C beamline in the Photon Factory of high energy accelerator research organization (KEK-PF). Each synchrotron radiation from the storage ring was monochromatized with Si(111) channel-cut crystals. The angle of the monochromator was calibrated using Pt foil. For transmission mode, ionization chambers filled with 15% Ar-85% N_2 mixed gas and 100% Ar were used as detectors to monitor the incident (I_0) and transmitted X-rays (I), respectively. For the fluorescence mode, a 7-element silicon-drift detector was employed. All the measurements were conducted at room temperature. XAFS analyses were conducted using REX2000 software (Rigaku Co. Japan).

3. Preparation of Fmoc-(E-Pt)-OH (1) used for SPPS method

Scheme S1.

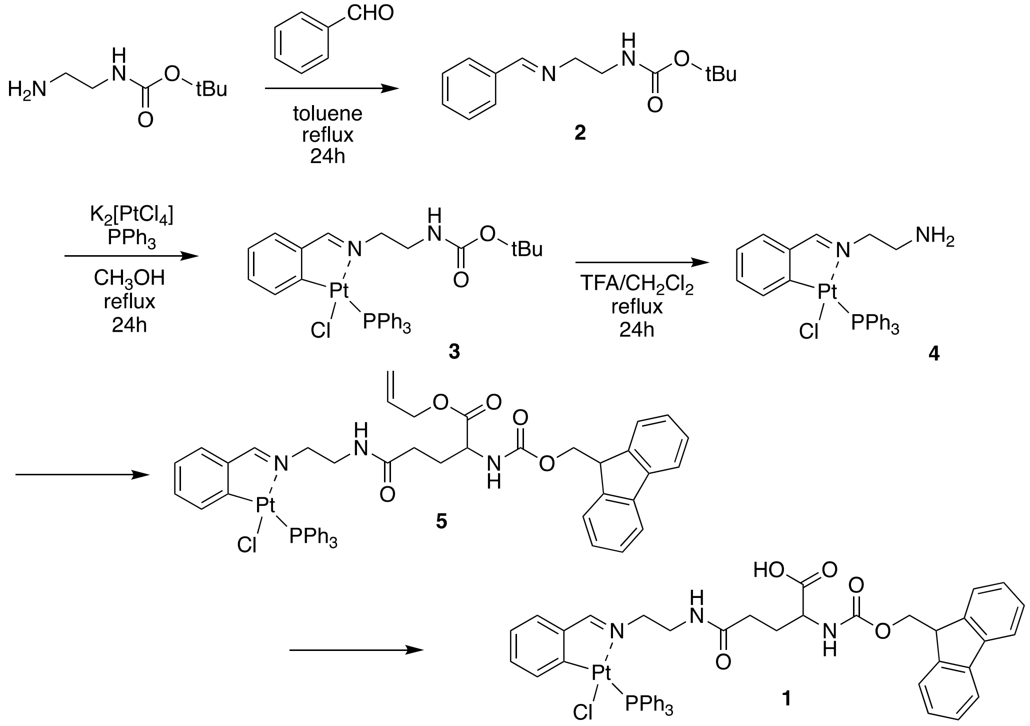

Synthesis of compound 2. N-(tert-Butoxycarbonyl)-1,2-diaminoethane (5.00 g, 31.2 mmol), benzaldehyde (3.48 g, 32.8 mmol), and 100 ml of dehydrated toluene were placed in a flask with a Dean-Stark apparatus and a reflux tube; the mixture was stirred at 115°C-125°C for 24 hours with reflux.

The appearance of the solution changed from cloudy white to transparent light yellow. After the reaction, the solution was concentrated to dryness, and the crude product of **2** (7.59 g, 98%) was confirmed by ^{1}H NMR ($CDCl_3$) and APCI-MS.

^{1}H NMR (400MHz, $CDCl_3$, 301K, δ in ppm) 8.28 (s, 1H, -CH=N-), 7.72 (d, J = 7.6 Hz, 2H, -C_6H_5), 7.41-7.32(m, 3H, -C_6H_5), 4.91(s, 1H, -NH-), 3.71(t, J = 5.6 Hz, 2H, -CH_2-), 3.47(t, J = 5.6 Hz, 2H, -CH_2-), 1.43(s, 9H, -C(CH_3)$_3$). ^{13}C NMR (100 MHz, $CDCl_3$, δ in ppm) 162.6, 155.9, 136.0, 130.8, 128.6, 128.2, 60.9, 41.4, 28.4. APCI-MS. m/z Calcd for $C_{14}H_{20}N_2O_2$ $[M+H]^+$ 249.16, Found 249.24.

Anal. calcd for $C_{14}H_{20}N_2O_2$: C, 67.72; H, 8.12; N, 11.28; O, 12.89, found : C, 67.39; H, 7.93; N, 11.28; O, 12.68.

Synthesis of compound 3. Crude **2** (4.18 g, 16.9 mmol), $K_2[PtCl_4]$ (7.00 g, 16.9 mmol), and 280 ml of dehydrated methanol were placed in a flask. It was stirred under argon with reflux for 24 hours in a light-shielded condition. After the reaction, the black suspension was filtered. Then, triphenylphosphine (4.42 g, 16.9 mmol) was added to the yellow filtrate and stirred under air for another 3 h. The reaction mixture was concentrated to remove the solvent. It was again dissolved in chloroform and filtered to remove the insoluble part. The solution was further purified by silica-gel column chromatography (hexane/ethylacetate = 1/1) to afford 400 mg of product **3** (2.17 g, 17%).
^{1}H NMR (400MHz, $CDCl_3$, 301K, δ in ppm) 8.24 (d, $J_{P\text{-}H}$ = 9.2 Hz, $J_{Pt\text{-}H}$ = 91.2 Hz, 1H, -C*H*=N-), 7.76-7.72(m, 6H, -P(C_6H_5)$_3$), 7.44-7.41(m, 3H, -P(C_6H_5)$_3$), 7.38-7.34 (m, 6H, -P(C_6H_5)$_3$), 7.31 (d, *J* = 7.2 Hz, 1H, -PtC_6H_4-) 6.91(dd, 1H, J = 7.2, 7.2 Hz, -PtC_6H_4-), 6.65-6.50 (m, 2H, -PtC_6H_4-), 4.96 (br, 1H, -N*H*-), 4.24(br, 2H, -CH_2-), 3.60 (td, *J* = 6.0, 6.4 Hz, 2H, -CH_2-), 1.43(s, 9H, -C(CH_3)$_3$). ^{13}C NMR (100 MHz, $CDCl_3$, δ in ppm) 179.0, 179.0, 156.1, 146.3, 145.2, 145.1, 137.2, 137.1, 135.5, 135.4, 131.7, 131.6, 130.8, 130.8, 130.4, 129.8, 128.2, 128.0, 127.9, 123.0, 79.3, 57.3, 41.2, 28.4. APCI-MS. *m*/*z* calcd for $C_{32}H_{34}ClN_2O_2PPt$ $[M+H]^+$ 740.18, found 740.37. Anal. calcd for $C_{32}H_{34}ClN_2O_2PPt$: C, 51.54; H, 4.63; Cl, 4.79; N, 3.78; O, 4.32, P, 4.18; Pt, 21.36, found : C, 51.54; H, 4.77; Cl, 4.78; N, 3.79.

Synthesis of compound 4. Product **3** (3.02 g, 4.08 mmol) in a flask was dissolved in a mixture of dichloromethane (6 mL) and trifluoroacetic acid (6 mL). The mixture was stirred for 5 minutes at room temperature. After the reaction, an excess amount of dichloromethane was added, and the reaction mixture was neutralized by adding an aqueous Na_2CO_3 solution. The solution was washed twice with Na_2CO_3 solution, water, and saline, respectively, and the organic layer was recovered and concentrated. The product was isolated by column chromatography (methanol/chloroform gradient from 0% to 20%) to afford **4** (2.34 g, 90%). Because the sample was moisture-sensitive, the crude product was used for the next step without further purification.
^{1}H NMR (400MHz, $CDCl_3$, 301K, δ in ppm) 8.30 (d, $J_{P\text{-}H}$ = 11.2 Hz, $J_{Pt\text{-}H}$ = 94.8 Hz, 1H, -C*H*=N-), 7.67-7.62 (m, 6H, -P(C_6H_5)$_3$), 7.48-7.42 (m, 9H, -P(C_6H_5)$_3$), 7.31 (d, *J* = 7.2 Hz, 1H, -PtC_6H_4-) 6.99 (dd, 1H, *J* = 7.2, 7.2 Hz, -PtC_6H_4-), 6.74 (dd, 1H, *J* = 7.2, 7.2 Hz, -PtC_6H_4-), 6.46 (d, $J_{H\text{-}H}$ = 7.2, 1H, -PtC_6H_4-), 4.53 (br, 1H, -NH_2), 4.14(br, 2H, -CH_2-), 3.35 (t, *J* = 5.6 Hz, 2H, -CH_2-). ^{13}C NMR (100 MHz, $CDCl_3$, δ in ppm) 177.0, 152.6, 145.0, 138.2, 135.5, 132.9, 132.6, 130.7, 130.4, 130.1, 130.0, 129.9, 125.3, 118.3, 58.1, 49.0. MALDI-TOF-MS. *m*/*z* Calcd for $C_{27}H_{26}ClN_2PPt$ $[M–Cl]^+$ 604.15, Found 604.55.

Synthesis of compound 5. Fmoc-L-glutamic acid 1-allyl ester (0.15 g, 0.37 mmol) and 1-(3-Dimethylaminopropyl)-3-ethyl carbodiimide (EDCI: 0.13 g, 0.68 mmol) were dissolved in 11 ml of CH_2Cl_2. Then, **4** and 4-Dimethylaminopyridine (DMAP: 7.3 mg, 0.06 mmol) were added and stirred for 24 hours at room temperature. The resulting solution was washed with HCl solution (1 mol L^{-1}), water, and saline twice each to remove EDCI and DMAP. The organic layer was

then purified by silica-gel column chromatography (ethyl acetate/ hexane gradient from 0% to 65%) to afford product **5** (0.23 g, 66%).

^{1}H NMR (400MHz, $CDCl_3$, 301K, δ in ppm) 8.27 (d, J_{P-H} = 8.8 Hz, J_{Pt-H} = 86.8 Hz, 1H, -C*H*=N-), 7.75-7.70 (m, 8H, -P(C_6*H_5*)$_3$, -CH(C_6*H_4*)$_2$), 7.59 (d, *J* = 8.4 Hz, 1H, -CH(C_6*H_4*)$_2$), 7.58 (d, *J* = 8.4 Hz, 1H, 1H, -CH(C_6*H_4*)$_2$), 7.43-7.27 (m, 14H, -P(C_6*H_5*)$_3$, -CH(C_6*H_4*)$_2$, -PtC_6*H_4*-), 6.85 (dd, 1H, *J* = 7.2, 7.2 Hz, -PtC_6*H_4*-), 6.74 (br, 1H, -PtC_6*H_4*-), 6.55-6.48 (m, 2H, -PtC_6*H_4*-), 5.93-5.80 (m, 3H, -CH_2N*H*CO-, -COOCH$_2$C*H*=CH$_2$, FmocN*H*-), 5.33 (d, 1H, J = 17.2 Hz, -COOCH$_2$CH=C*H$_2$*), 5.24 (d, *J* = 10.4 Hz, 1H, -COOCH$_2$CH=C*H$_2$*), 4.61 (d, *J* = 5.2 Hz, 2H, -COOC*H$_2$*CH=CH$_2$), 4.42-4.17 (m, 6H, =NC*H$_2$*CH$_2$NH, FmocNHC*H*-, -C*H$_2$*CH(C_6H_4)$_2$, -C*H*(C_6H_4)$_2$), 3.73-3.70 (m, 2H, =NCH$_2$C*H$_2$*NH,), 2.31-2.15 (m, 3H, –CHC*H$_2$*CH$_2$CO-, –CHCH$_2$C*H$_2$*CO-), 2.01 (br, 1H, –CHC*H$_2$*CH$_2$CO–). ^{13}C NMR (100 MHz, $CDCl_3$, δ in ppm) 179.6, 172.4, 171.7, 156.2, 146.1, 144.9, 144.0, 143.8, 141.3, 137.1, 135.5, 131.8, 131.6, 130.9, 130.3, 129.7, 128.5, 128.0, 127.7, 127.1, 125.2, 123.2, 120.0, 119.0, 67.0, 66.1, 56.1, 53.8, 47.2, 41.3, 32.2, 27.8. MALDI-TOF-MS. *m/z* calcd for $C_{49}H_{45}ClN_3O_5PPt$ [M–Cl]$^+$ 995.29, found 996.92. Anal. calcd for $C_{50}H_{47}ClN_3O_5PPt$: C, 58.22; H, 4.59; Cl, 3.44; N, 4.07; O, 7.76; P, 3.00; Pt, 18.91, found : C, 57.87; H, 4.38; Cl, 3.59; N, 3.62.

Synthesis of compound 1. Compound **5** (804 mg, 0.78 mmol), Tetrakis(triphenylphosphine)palladium (Ph(PPh_3)$_4$: 90 mg, 0.078 mmol) , and Dimedone (242 mg, 1.73 mmol) were dissolved in 80 mL of CH_2Cl_2 and stirred under argon for 3 hours at room temperature. As the reaction progressed, the solution color changed from yellow to orange. The product was purified by silica gel column chromatography (methanol/chloroform gradient from 0% to 10%) to afford product **1** (580 mg, 75%).

Because compound **1** tends to form self-assemblies in solution, the characterization based on ^{1}H NMR spectrum with broad peaks was difficult. Here, the products were confirmed only by MALDI-TOF-MS. Also, the purity was determined to be 99.6 % by analytical HPLC (UV detector at 214 nm).

MALDI-TOF-MS. *m/z* Calcd for $C_{47}H_{43}ClN_3O_5PPt$ [M – Cl]$^+$ 955.26, Found 956.61.

4. Preparation of Fmoc-(K-Fe)-OH (6) used for SPPS method

Scheme S2.

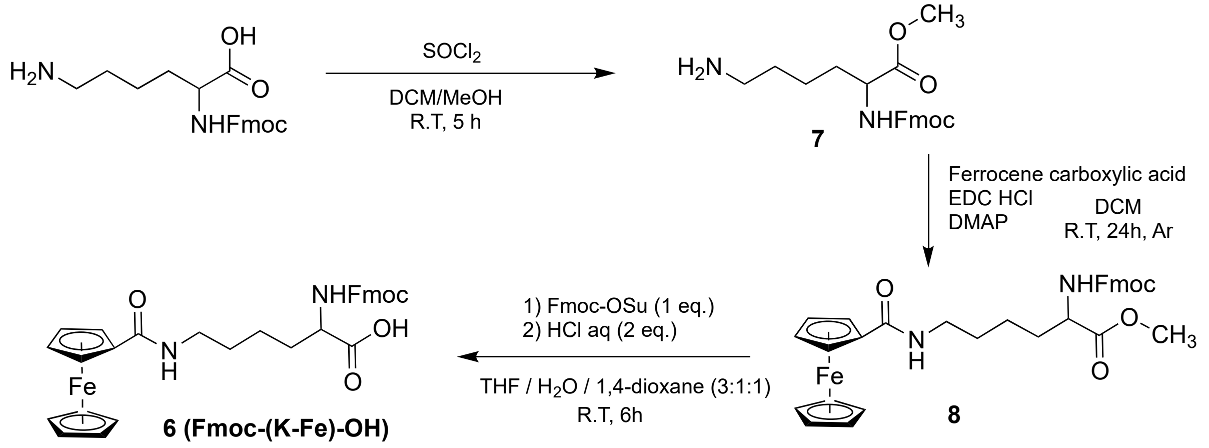

Synthesis of compound 8. Fmoc-Lys(Boc)-OH 500 mg (1.07 mmol) was dissolved in a DCM/MeOH solution (15 ml/135 ml) and cooled to about 0°C using an ice bath. After sufficient cooling, the solution was stirred and $SOCl_2$ 200 µl (2.76 mmol) was gradually added. The mixture was stirred in the ice bath for 1 hour, then allowed to return to room temperature, followed by additional stirring for 4 hours. The solution was concentrated under reduced pressure and the minimum amount of MeOH necessary to dissolve the precipitated white powder was added to recover a solution. Excess diethyl ether was then added and the mixture was placed in a freezer for one day. Re-precipitated white powder of **7** was collected, yielding 232.6 mg (57%).

In a 25 mL Schlenk tube, Ferrocene carboxylic acid (94.0 mg, 1.2 equiv.), and the condensing agent EDC HCl (127 mg,1.2 equiv.) were added, and the system was purged with Ar. About 6 mL of anhydrous CH_2Cl_2 was added, and the mixture was stirred. After confirming that the carboxylic acid and the condensing agent were completely dissolved and the solution became clear, **7** (125 mg, 1 equiv.) and the condensation co-agent DMAP 12.0 mg (0.3 equiv.) were added to initiate the reaction. The reaction was carried out at room temperature for 24 hours, and after completion, the solvent was removed under reduced pressure. Subsequently, the product was purified using silica gel column chromatography (Hexane / Chloroform = 1/1 v/v – Hexane / Chloroform = 0/1 v/v – Chloroform / Methanol = 97/3 v/v). The target compound (**8**) was obtained as an orange powder (133 mg, 69%).

^{1}H-NMR ($CDCl_3$, 400 MHz, 301K, δ in ppm) 1.45-1.51 (m, 2H, -$COCHCH_2CH_2$-), 1.62 (t, 2H, *J* = 12.4 Hz, -$COCHCH_2CH_2CH_2$-), 1.75-1.93 (m, 2H, -$COCHCH_2$-), 3.39 (q, 2H , *J* = 6.2 Hz, $(C_5H_5)Fe(C_5H_5)CONHCH_2$-), 3.76 (s, 3H, -$OCH_3$), 4.17 (s, 5H, $(C_5H_5)Fe$-), 4.23 (t, 1H, *J* = 7.0 Hz, FmocNHC*H*-), 4.29 (s, 2H, $(C_5H_5)Fe(C_5H_5)CO$-), 4.34-4.45 (m, 3H, $(C_6H_4)_2CH$-, $(C_6H_4)_2CHCH_2$-), 4.66 (d, 2H, *J* = 9.6 Hz, $(C_5H_5)Fe(C_5H_5)CO$-), 5.51 (d, 1H, *J* = 7.6 Hz, FmocN*H*-), 5.77 (s, 1H, $(C_5H_5)Fe(C_5H_5)CONH$-), 7.31 (t, 2H, *J* = 7.4 Hz, $(C_6H_4)_2CH$-), 7.40 (t, 2H, *J* = 7.5 Hz, $(C_6H_4)_2CH$-), 7.61 (d, 2H, *J* = 7.4 Hz, $(C_6H_4)_2CH$-), 7.77 (d, 2H, *J* = 7.5 Hz, $(C_6H_4)_2CH$-). ^{13}C NMR (100 MHz, $CDCl_3$, δ in ppm) 22.4(1C, $CH_3OCOCHCH_2CH_2$-), 29.3(1C, $CH_3OCOCHCH_2$-), 32.2(1C, $CH_3OCOCHCH_2CH_2CH_2$-), 39.0(1C, $(C_5H_5)Fe(C_5H_5)CONHCH_2$-), 47.2(1C, $(C_6H_4)_2CH$-), 52.5(1C, CH_3O-), 53.6(1C, CH_3OCOCH-), 67.1(1C, $(C_6H_4)_2CHCH_2$-), 68.0(1C, $(C_5H_5)Fe(C_5H_5)$-), 68.2 (2C, $(C_5H_5)Fe(C_5H_5)$-), 69.7(5C, $(C_5H_5)Fe(C_5H_5)$-), 70.4(2C, $(C_5H_5)Fe(C_5H_5)$-), 120.0(2C, $(C_6H_4)_2CH$-), 125.1(2C, $(C_6H_4)_2CH$-), 127.1(2C, $(C_6H_4)_2CH$-), 127.7(2C, $(C_6H_4)_2CH$-), 141.3(2C, $(C_6H_4)_2CH$-), 143.8(1C, $(C_6H_4)_2CH$-), 143.9(1C, $(C_6H_4)_2CH$-), 156.1(1C, $(C_6H_4)_2CHCH_2OCO$-), 170.4(1C, CH_3OCO-), 173.0(1C, $(C_5H_5)Fe(C_5H_5)CO$-). MALDI-TOF-MS (DCTB, $CHCl_3$) *m/z* calcd for $C_{33}H_{34}FeN_2O_5$ $[M]^+$ 594.49, found 594.57. Anal. calcd for $C_{33}H_{34}FeN_2O_5$: C 66.67, H 5.76, Fe 9.39, N 4.71, O 13.46, found: C 66.58, H 6.05, N 4.62.

Synthesis of compound 6. 8 (133 mg, 1 equiv.) was dissolved in 15 mL of THF/H_2O/1,4-dioxane (3:1:1) and stirred at 0°C. To this solution, 0.05 M LiOH aqueous solution was added dropwise in 3.0 mL increments every 10 minutes, three times. The mixture was then stirred for 1 hour at 0°C and for 1.5 hours at room temperature. Subsequently, *N*-(9-Fluorenylmethoxycarbonyloxy)succinimide
(Fmoc-OSu) (1.2 equiv.) was added to the reaction solution and stirred at room temperature for 6 hours. After the reaction was completed, aqueous HCl was added to neutralize the mixture,

and $CHCl_3$ was added followed by washing twice with aqueous NH_4Cl. The organic layer was collected and dried over $MgSO_4$. After removing the solvent under reduced pressure, the product was purified using silica gel column chromatography (Chloroform / Methanol = 1/0 → 9/1 v/v). The target compound **9** was obtained as an orange powder (102 mg, 78%).
^{1}H-NMR ($CDCl_3$, 400 MHz, 301K, δ in ppm) 1.34-1.67 (m, 4H, -COCHCH_2CH_2-), 1.69-2.04 (m, 2H, -COCHCH$_2$CH$_2$CH_2-), 3.34 (s, 2H, (C$_5$H$_5$)Fe(C$_5$H$_5$)CONHCH_2-), 4.14 (s, 6H, (C$_5$$H_5$)Fe-, FmocNHC$H$-), 4.21-4.49 (m, 5H, (C$_6H_4$)$_2CH$-, (C$_6H_4$)$_2CHCH_2$-, (C$_5H_5$)Fe(C$_5$$H_5$)-), 4.69 (s, 2H, (C$_5H_5$)-Fe-(C$_5$$H_5$)-), 5.94 (s, 1H, FmocN$H$-), 6.23 (s, 1H, (C$_5H_5$)Fe(C$_5H_5$)CON$H$-), 7.25 (t, 2H, J = 6.4 Hz, (C$_6$$H_4$)$_2$CH-), 7.35 (t, 2H, J = 13.8 Hz, (C$_6$$H_4$)$_2$CH-), 7.56 (s, 2H, (C$_6$$H_4$)$_2$CH-), 7.71 (d, 2H, J = 7.4 Hz, (C$_6$$H_4$)$_2$CH-), 8.51 (s, 1H, -COO$H$). ^{13}C NMR (100 MHz, $CDCl_3$, δ in ppm) 22.3(1C, HOCOCHCH$_2$CH$_2$-), 29.3(1C, HOCOCHCH$_2$-), 32.0(1C, HOCOCHCH$_2$CH$_2$CH$_2$-), 39.2(1C, (C$_5$H$_5$)Fe(C$_5$H$_5$)CONHCH$_2$-), 47.1(1C, (C$_6$H$_4$)$_2$CH-), 53.7(1C, HOCOCH-), 67.2(1C, (C$_6$H$_4$)$_2$CHCH$_2$-), 68.1(1C, (C$_5$H$_5$)Fe(C$_5$H$_5$)-), 68.3(2C, (C$_5$H$_5$)Fe(C$_5$H$_5$)-), 69.8(5C, (C$_5$H$_5$)Fe(C$_5$H$_5$)-), 70.6(2C, (C$_5$H$_5$)Fe(C$_5$H$_5$)-), 120.0(2C, (C_6H$_4$)$_2$CH-), 125.2(2C, (C_6H$_4$)$_2$CH-), 127.1(2C, (C_6H$_4$)$_2$CH-), 127.7(2C, (C_6H$_4$)$_2$CH-), 141.3(2C, (C_6H$_4$)$_2$CH-), 143.8(1C, (C_6H$_4$)$_2$CH-), 143.9(1C, (C_6H$_4$)$_2$CH-), 156.4(1C, (C$_6$H$_4$)$_2$CHCH$_2$OCO-), 171.5(1C, HOCO-), 174.5(1C, (C$_5$H$_5$)Fe(C$_5$H$_5$)CO-). MALDI-TOF-MS (DCTB, $CHCl_3$) m/z calcd for $C_{32}H_{32}FeN_2O_5$ $[M]^+$ 580.46, found 580.68. Anal. calcd for $C_{32}H_{32}FeN_2O_5$: C 66.21, H 5.56, Fe 9.62, N 4.83, O 13.78, found: C 65.92, H 5.81, N 4.74.

5. Synthesis of H-(E-Pt)$_n$-OH (n = 1, 2, 3, 4, 6, 12)
The platinum-containing peptide oligomers (**H-(E-Pt)$_n$-OH**) were synthesized using an automatic peptide synthesizer (Biotage Initiator). Trityl-OH ChemMatrix (Sigma Aldrich) was used for the SPPS resin. Typical synthetic procedure at 0.015 mmol scale is described below.

25 mg of the resin was placed in a 10 ml reaction tube, and the resin was swollen by adding CH_2Cl_2 and shaking for 3 hours. Then, 2 ml of 2% $SOCl_2$ dichloromethane solution was added and shaken overnight. The resin was then washed five times with dichloromethane and three times with 2% Diisopropylethylamine dichloromethane solution to obtain the activated resin.

The reaction tube containing the activated resin was installed in an automatic synthesizer, and the following condensation and deprotection steps were repeated for a specified number of times. Finally, the solid phase cleavage was performed.

A. Condensation: The reaction was carried out at 75°C (microwave heating) for 5 minutes by adding the following reagents.
- HATU (0.02 mmol) in DMF
- HoAt (0.02 mmol) in DMF
- *N*-ethyl-diispropylamine (0.02 mmol)
- **1** (0.05 mmol) in DMF

B. Deprotection: The reaction was carried out at 75°C (microwave heating) for 10 minutes by adding the following reagent.
- 20 % Piperidine in DMF

C. Cleavage: The reaction tube was removed from the automatic synthesizer. Then, the following solution was added.

- DCM : TFA : TIPS = 96.5 : 1.0 : 2.5

After 1 hour, the precipitation afforded by adding cold diethyl ether to the eluent was collected by centrifugation. The total yields of product after the freeze-drying was 5.3 mg (7.5%, 13 steps) and 0.45 mg (0.32%, 25 steps) for **H-(E-Pt)$_6$-OH** and **H-(E-Pt)$_{12}$-OH**, respectively.

6. Synthesis of H-(K-Fe)$_n$-OH (n = 12)

The iron-containing peptide oligomers (**H-(K-Fe)$_n$-OH**) was synthesized using an automatic peptide synthesizer (Biotage Initiator). Rinkamide-trt(2-Cl) Resin (Watanabe Chemical Industry Inc) was used for the SPPS resin. Typical synthetic procedure at 0.68 mmol scale is described below.

40 mg of the resin was placed in a 10 ml reaction tube, and the resin was swollen by adding DCM and shaking for 3 hours. The resin was then washed five times with dichloromethane and three times with 2% DIEA / DCM solution to obtain the activated resin.

The reaction tube containing the activated resin was installed in an automatic synthesizer, and the following condensation and deprotection steps were repeated for a specified number of times. Finally, the solid phase cleavage was performed.

A. Condensation: The reaction was carried out at 75°C (microwave heating) for 5 minutes by adding the following reagents.

- HATU (0.0816 mmol, 3.0 equiv.) in DMF
- HoAt (0.0789 mmol, 2.9 equiv.) in DMF
- DIEA (0.163 mmol, 6.0 equiv)
- **6** (0.0816 mmol, 3.0 equiv.) in DMF

After the condensation was complete, capping of unreacted sites on the resin was performed by adding 250 μL of anhydrous acetic acid, 250 μL of DIEA, and 500 μL of DMF, followed by stirring at room temperature for 15 minutes.

B. Deprotection: The reaction was carried out twice at room temperature for 5 minutes and 15 minutes by adding the following reagent.

- 20 % Piperidine in DMF

C. Cleavage: The reaction tube was removed from the automatic synthesizer. Then, the following solution was added.

- DCM : TFA : TIPS : TFE = 66.5 : 1.0 : 2.5 : 30.0

To precipitate the peptides, *tert*-butylethylther was added to the filtrate. The crude product was obtained by centrifugation at 0°C for 20 minutes, followed by decanting. The crude product was purified by preparative reverse-phase HPLC, yielding 8.9 mg (7.1%) of the desired product.

7. Adsorption of Pt metallopeptides on Ketjenblack

Platinum metallopeptides (**H-(E-Pt)$_n$-OH**) obtained by SPPS were supported on high specific surface area carbon (Ketjenblack) as described below. It was used for the ADF-STEM observation by ADF-STEM and converted into metal subnanoparticles (**Pt$_n$**).

A 12-residue peptide (**H-(E-Pt)$_{12}$-OH**) is described as an example: 20 μg of **H-(E-Pt)$_{12}$-OH** was dissolved in 2 mL of chloroform; 1.0 mg of Ketjenblack (EC600JD) was placed in 2 mL of chloroform and allowed to disperse by ultrasonication for 15 min. Then, it was mixed homogenously with a chloroform solution of the peptide. This process was scaled up as needed.

8. Chemical conversion of Pt metallopeptides to Pt subnanoparticles

The powder of platinum metallopeptide (**H-(E-Pt)$_n$-OH**) supported on carbon was placed in a glass reaction vessel and treated under hydrogen gas stream (3% H_2/N_2, 3 L min^{-1}) at 250 °C in a tube furnace (KOYO Thermosystem Inc, KTF040N1-AS) for 3 hours. Basically, the same conditions are used for all residue numbers. The products were used for subsequent ADF-STEM observations, XPS measurements, and catalytic activity evaluation.

9. ADF-STEM observation of metallopeptides and Pt subnanoparticles

Carbon-supported metallopeptides and platinum subnanoparticles were sampled and observed as follows. A very small amount of carbon-supported samples were ultrasonically dispersed in methanol and drop-cast onto an elastic carbon film with a Cu mesh (Nisshin EM Co.) followed by vacuum drying at 40 °C overnight.

ADF-STEM images were acquired at room temperature using an 80 kV cold-FEG TEM (JEOL, JEM-ARM200F) with spherical aberration-corrected probe (CEOS, ASCOR). Unless otherwise specified in the figure captions, a convergence angle was 31.8 mrad, and the inner and outer collection angles used for the ADF images were 57 and 226 mrad, respectively.

Computational simulations of STEM images were performed using Bionet elbis software (Bionet Laboratory Inc.) at accelerating voltage of 80 kV, defocus value of 2-5 nm, and spherical aberration coefficient of 1 μm. The inner and outer collection angles used in simulating the ADF images were 57 and 226 mrad, respectively. Conformation search of **H-(E-Pt)$_{12}$-OH** suitable for the observed ADF-STEM images started at the model structure where 12 monomers were linearly connected. Dihedral angles around C-C and C-N bonds in the main chain and orientations of the side chains were modified to match simulated images to the observed images. No overlapping of atoms in the optimized structures was confirmed.

10. EXAFS data analysis

According to the ADF-STEM image of **Pt_{12}/KB** (**Fig. S8**), one Pt atom is adjacent to other Pt atoms and they are aggregated as a single particle. The particles are relatively flat and in contact with the underlying carbon support. Furthermore, the XPS (**Fig. S7**) and XANES (**Fig. S9a**) reveal that the valence states of the Pt atoms are between zero and mono-valent. The curve fitting of EXAFS is aimed at verifying whether the experimental results are consistent with a model proposed by the ADF-STEM images.

Here, we assume a model in which one Pt atom is adjacent to several other Pt atoms and light elements such as C, O, and N. If the Pt atom is zero-valent, the degeneracy of the first proximity should be 12. However, we set the degeneracy constraint to be less than 10 because partial oxidation was confirmed by the XPS. In the previous report on Pt_{12} cluster[1], the degeneracy of Pt-Pt was about 4-6, which was determined by EXAFS measured at low temperature (30 K). The maximum of the degeneracy of Pt-C is 6 when Pt atoms are bonded to hexagonal sites in the graphite structure. Therefore, the constraint of total degeneracy being less than 10 is reasonable.

First, we analyzed Pt_{12}/KB synthesized by an established dendrimer template method[1], for which relatively high *s/n* XAFS data was obtained by the transmission method. The FT-EXAFS exhibited only a Pt-L (L is C, N or O) shell that appeared at 1.5-2.2 Å, whereas a Pt-Pt shell appeared at 2.2-3.0 Å is significantly attenuated. Therefore, a curve-fitting analysis with the Pt-L only single-shell model was performed (**Fig. S9c**). The *R*-factor of the curve fitting (CF) was 11%, and the degeneracy including the error was 8.6 ± 1.2, and the interatomic distance was 2.17 Å. Given that this is a much longer interatomic distance than the usual Pt-C, Pt-O or Pt-N bonds found in the metal complexes, the present data of EXAFS are consistent with the model, assuming a weak interaction with the support as described. However, the comparison between the experimental Fourier-transform EXAFS and the fitting curve shows that the curve-fitting (CF) accuracy is unsatisfactory because the contribution from a weak but definitely heavy scattering atoms exists at 2.2~3.0 Å.

Then, a further CF was performed by adding a Pt-Pt shell as a heavy scattering atom to the previous curve-fitting model (**Fig. S9d**). In this case, the *R*-factor was reduced to 3.8%, which is more consistent with the experimental results when Pt-Pt is taken into account compared to the Pt-*L* only model. The degeneracy of Pt-Pt shell in the two-shell CF was 1.4, and the interatomic distance was 2.74 Å, which is consistent with the previous result measured at low temperature (30 K).[1]

On the other hand, the signal-to-noise ratio of the peptide-based Pt_{12}/KB measured by fluorescence method at room temperature was lower than that of the dendrimer-based Pt_{12}/KB measured by transmission method, making the CF difficult. Therefore, as qualitative and preliminary analysis, we compared the XANES of peptide-based **Pt_{12}/KB** and dendrimer-based **Pt_{12}/KB**. The XANES of the peptide-based **Pt_{12}/KB** and dendrimer-based **Pt_{12}/KB** were identical, indicating that they are in the same electronic state, and the EXAFS results are consistent with another one, although the signal-to-noise ratio is significantly different. These results suggest

that the peptide-based **Pt_{12}/KB** forms Pt subnanoparticles similar to those of the dendrimer-based **Pt_{12}/KB**.

11. Catalytic oxidation of toluene

The catalytic activity for the oxygen oxidation of toluene was evaluated using platinum supported on carbon. The experimental conditions are basically the same as previously reported.[2] The ^{1}H NMR spectra of the oxidative products exhibited the signals of benzyl alcohol (δ 4.63), benzaldehyde (δ 10.0), benzoic acid (δ 8.13), and benzyl benzoate (δ 5.37) in chloroform-d_1. The yields are estimated by comparison of the intensity ratio of these signals per the amount of the metal atoms on the basis of the signal of anisole (δ 3.81) added as an internal standard (**Table S1**).

12. Evolved gas thermogram

The evolved gas thermogram was recorded on a GC/MS instrument (Shimadzu, GCMS-QP2020 NX) equipped with a Multi-Shot Pyrolyzer (Frontier Lab, EGA/PY-3030D). Under the gas flow (He 160 mL/min + H_2 5 mL/min), the temperature of pyrolyzer was elevated from 40°C to 400°C at 10°C/min.

References

1 T. Imaoka, H. Kitazawa, W.-J. Chun, S. Omura, K. Albrecht and K. Yamamoto, *J. Am. Chem. Soc.*, 2013, **135**, 13089−13095.

2 M. Huda, K. Minamisawa, T. Tsukamoto, M. Tanabe and K. Yamamoto, *Angew. Chem. Int. Ed.*, 2019, **58**, 1002–1006.

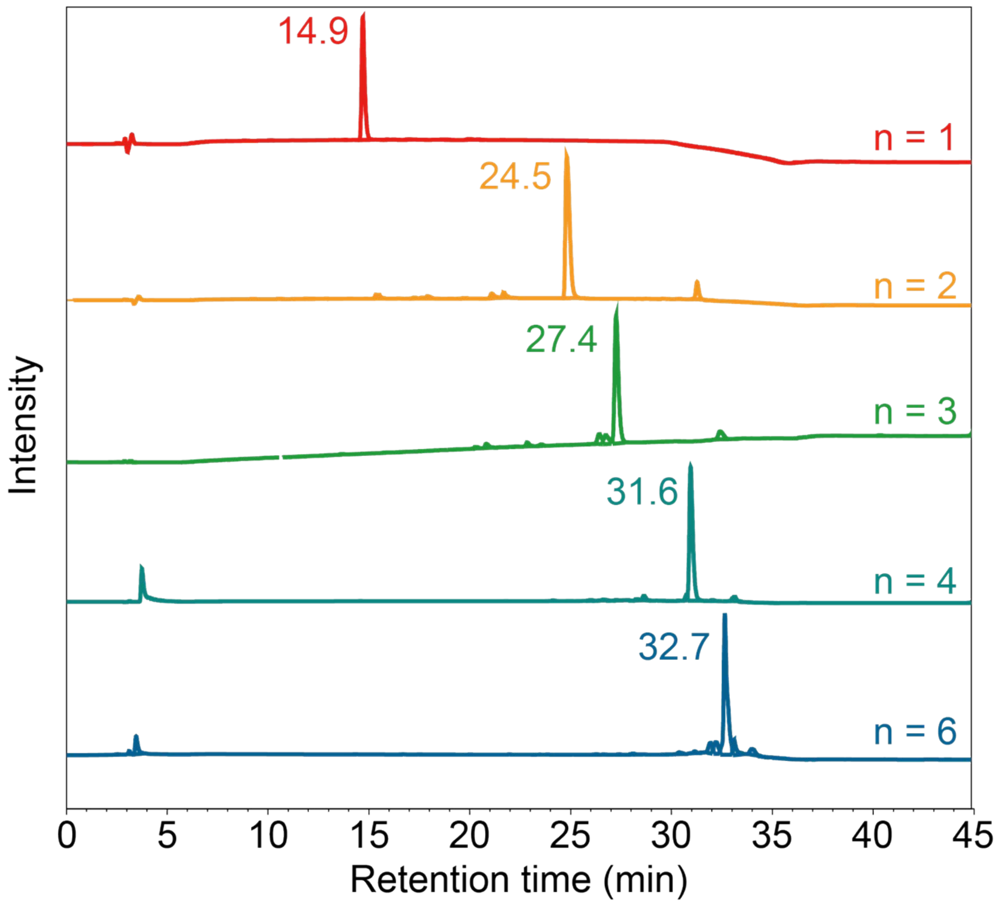

Fig. S1 Reversed-phase analytical HPLC charts of **H-(E-Pt)$_n$-OH** (n = 1, 2, 3, 4, 6) synthesized by the SPPS method. The solvent CH_3CN/H_2O (with 0.1 % TFA) was changed from 30% to 95%.

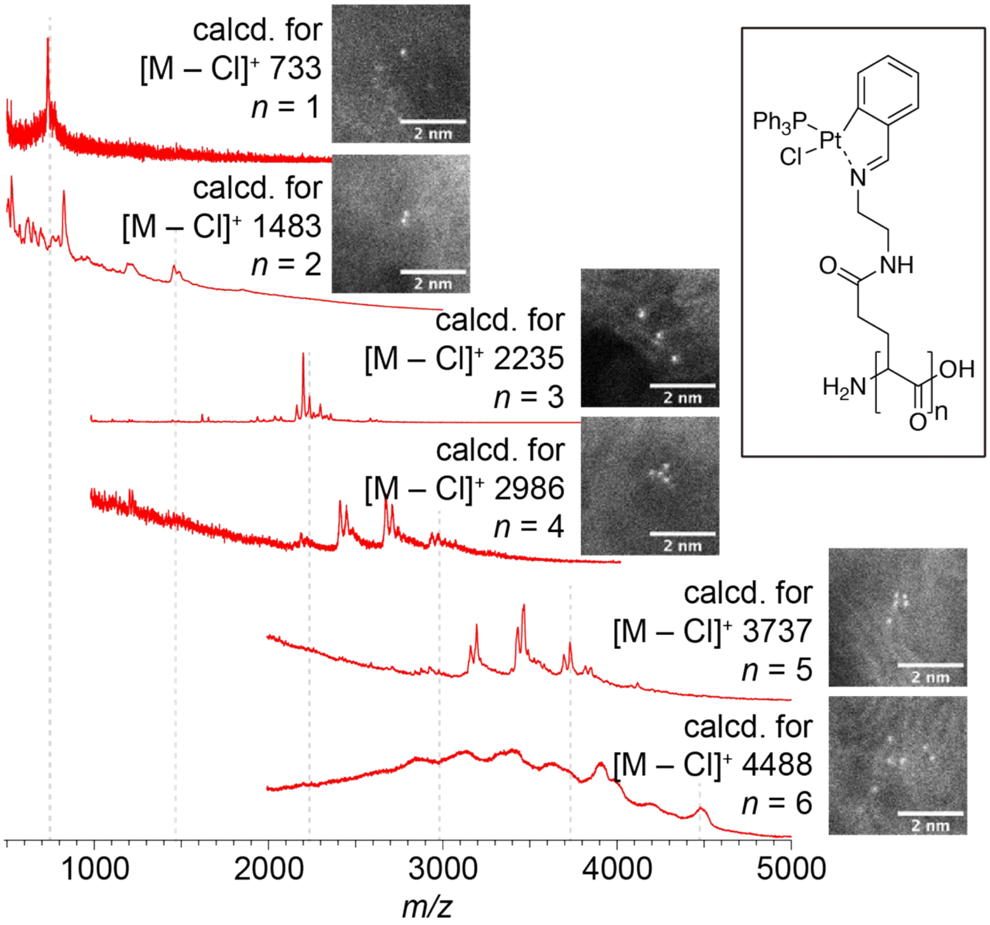

Fig. S2 MALDI-TOF-MS and atomic-resolution HAADF-STEM images of **H-(E-Pt)$_n$-OH (*n* = 1~6)** synthesized by the SPPS method.
DCTB (*trans*-2-[3-(4-tert-Butylphenyl)-2-methyl-2-propenylidene]malononitrile) was used for the
Matrix of MALDI-TOF-MS. The HAADF-STEM images of **H-(E-Pt)$_n$-OH (*n* = 1~6)** supported on Ketjenblack were collected with the convergence angle of 31.8 mrad and the inner and outer collection angles 57 and 226 mrad, respectively.

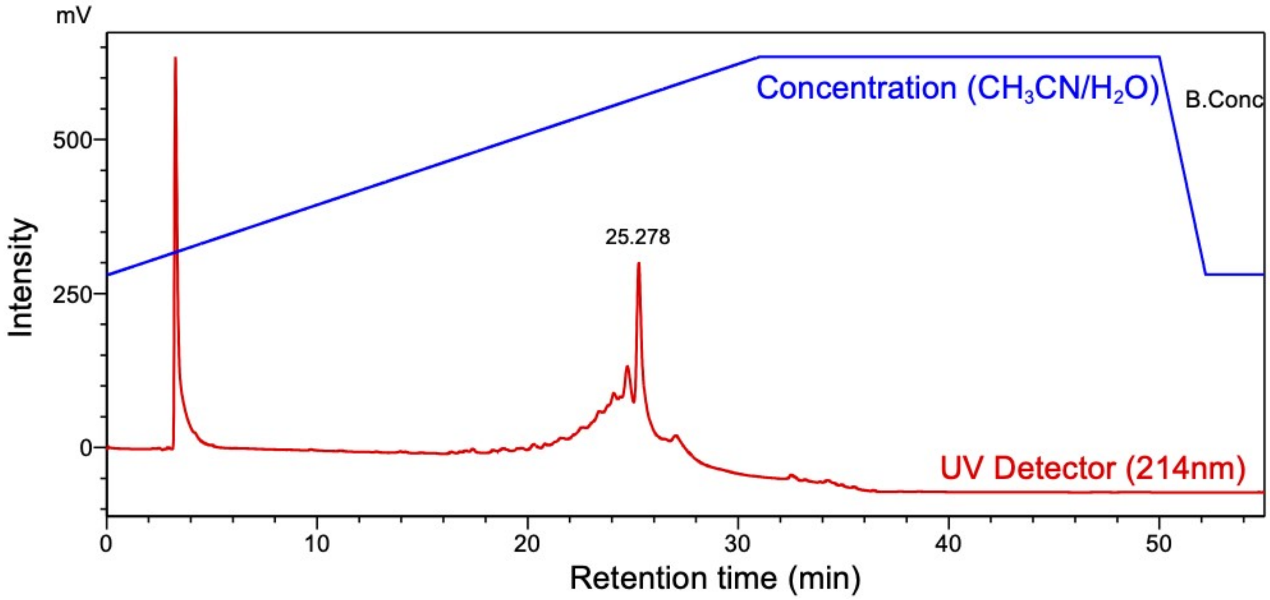

Fig. S3 Reversed-phase analytical HPLC charts of **H-(E-Pt)$_{12}$-OH** synthesized by the SPPS method. (Red) A chromatogram detected at 214 nm. (Blue) The ratio of the mixed solvent CH_3CN/H_2O (with 0.1 % TFA) was changed from 50% to 95%.

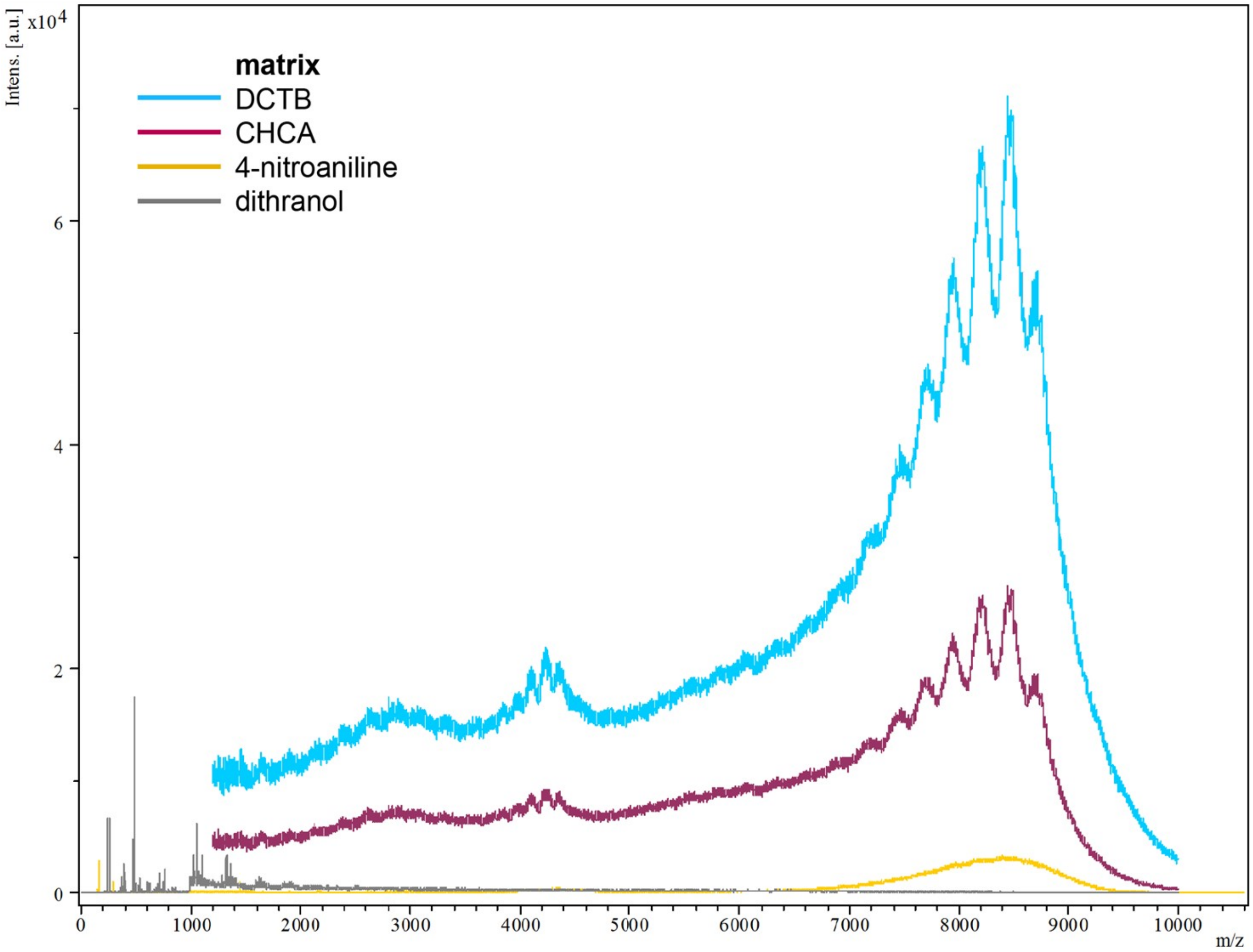

Fig. S4 MALDI-TOF-MS of **H-(E-Pt)$_{12}$-OH** synthesized by the SPPS method. Different matrixes, including DCTB (*trans*-2-[3-(4-tert-Butylphenyl)-2-methyl-2-propenylidene]malononitrile), CHCA (α-cyano-4-hydroxycinnamic acid), 4-nitroaniline or dithranol, was used for the matrix.

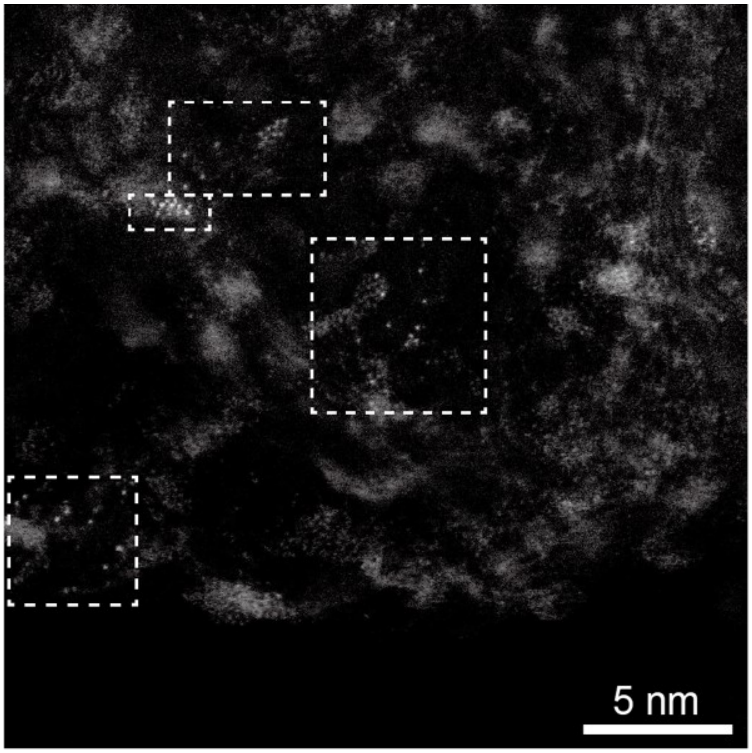

Fig. S5 An ADF-STEM image of **H-(E-Pt)$_{12}$-OH** supported on Ketjenblack. The 12 bright spots derived from the platinum complexes bound as side chains of the peptides are visible within the dotted rectangle.

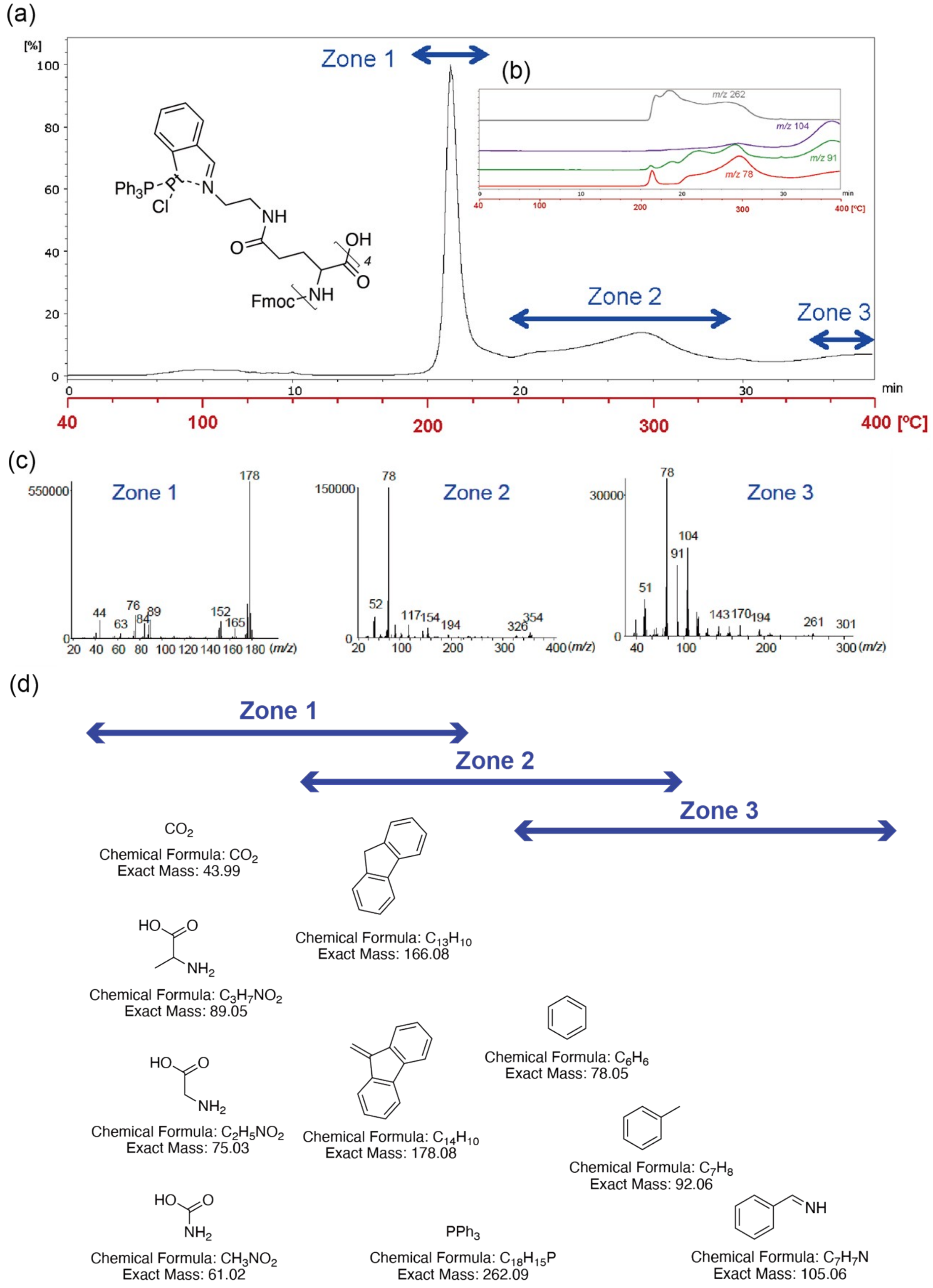

Fig. S6 (a) Total evolved gas thermogram of **Fmoc-(E-Pt)$_4$-OH**, (b) 2D mass chromatograms for different m/z, (c) Time-averaged mass spectra for different zones (Zone 1-3). (d) Possible assignments of the fragments observed in the mass spectra for different zones. According to the

analysis, the main components of the gas generated at temperatures lower than 250°C are fluorene derived from the Fmoc group and decomposition products of amino acids.

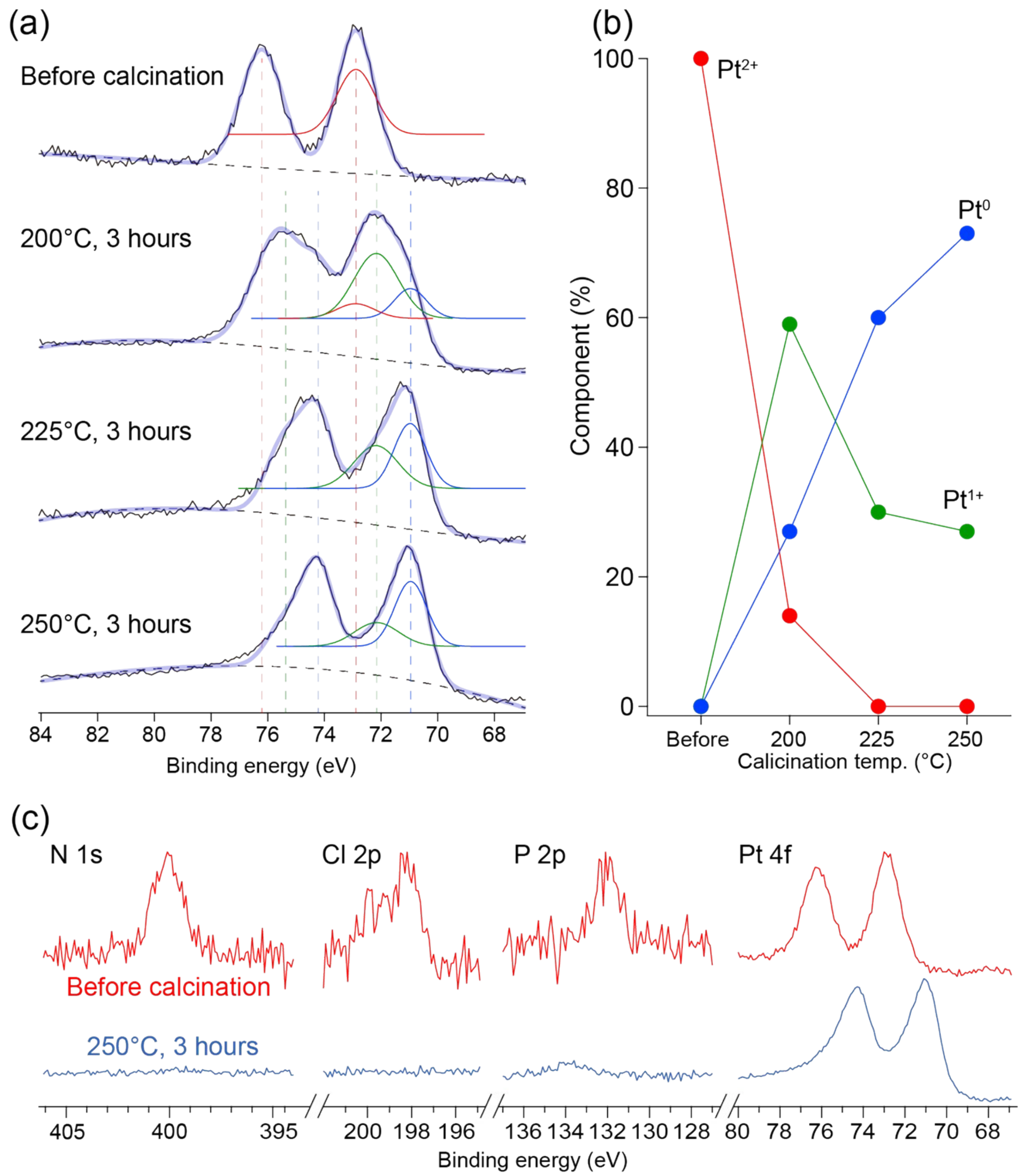

Fig. S7 XPS data of **H-(E-Pt)$_4$-OH** before and after the calcination under 3% H_2/N_2 gas flow. (a) The experimental (black line) and curve-fitting data (purple line) of Pt 4*f* peaks. Each component (red: Pt^{2+}, green: Pt^{1+}, red: Pt^0 valence states) is displayed for the Pt 4*f* 5/2 peak. (b) The amounts of respective components after the calcination for each 3 hours at different temperature. (c) Comparisons of the spectra at Pt 4*f*, P 2*p*, Cl 2*p* and N 1*s* peaks. The intensity of each spectrum is normalized so that the peak area of Pt 4*f* is equal.

(Note) The multipeak curve-fitting was carried out using Igor Pro 8 software. Each profile was treated as a Gaussian-type function with an invariant peak width and peak position, and only the peak intensity was used as the variable.

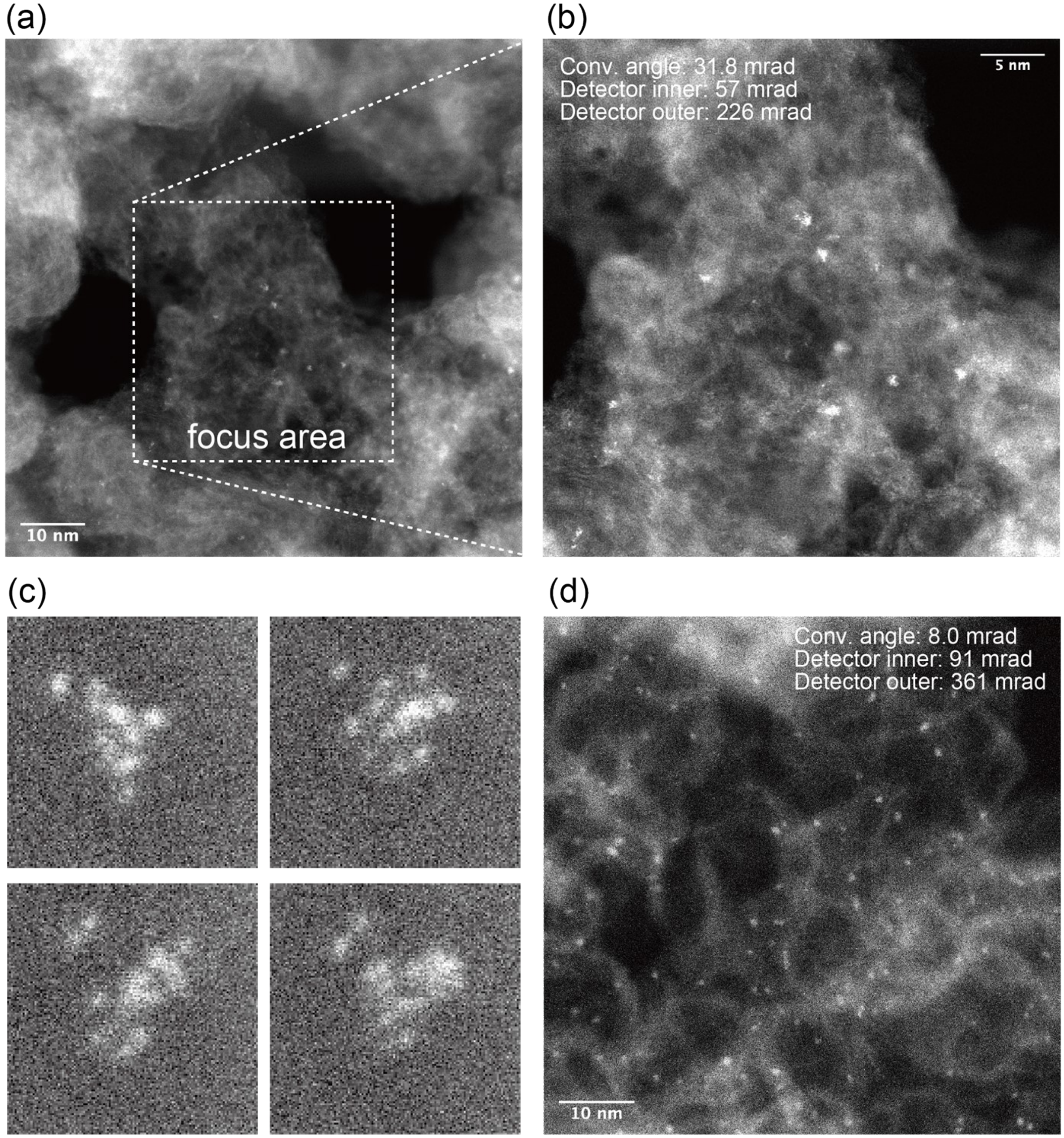

Fig. S8 ADF-STEM images (80 kV) of Pt_{12}/KB. (a) A low-magnification atomic-resolution image identical to that shown in **Fig. 3a**. Subnanoparticles are visible only at the focus area due to the very thin focus depth. The focused area is shown with a dashed circle. The image was collected with the convergence angle of 31.8 mrad and the inner and outer collection angles 57 and 226 mrad, respectively. (b) A higher magnification image at the focused area of (a). (c) Enlarged images of a single subnanoparticle cut out from an atomic-resolution video clip (**Video S1**). The length of one side of each image is 2.08 nm. It shows the fluidity of the atoms inside the subnanoparticle. (d) A pan-focused image for the visualization of subnanoparticles at different z-positions. The image was collected with the convergence angle of 8.0 mrad and the inner and outer collection angles 91 and 361 mrad, respectively.

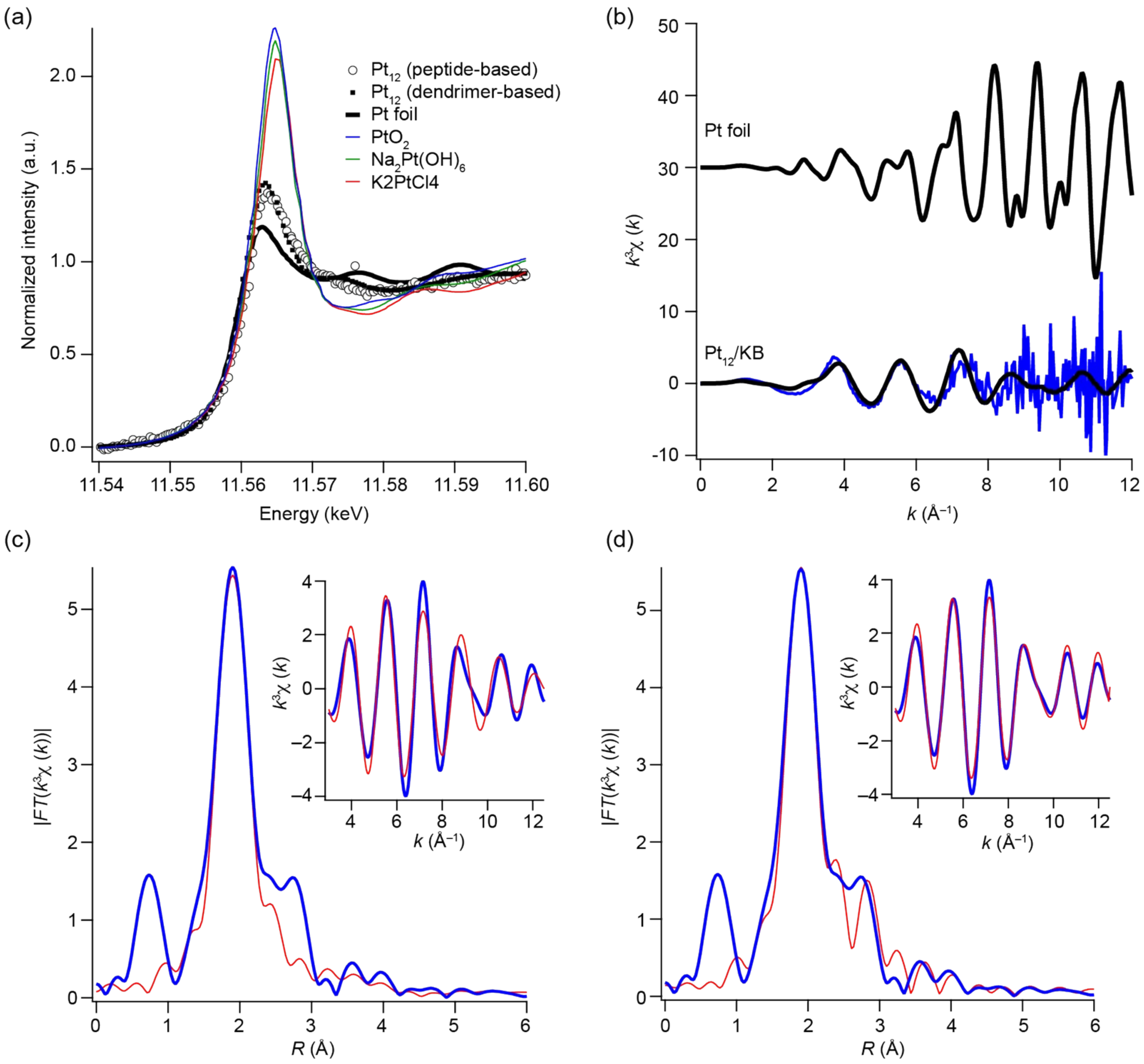

Fig. S9 Pt-L_3 edge XAFS. Pt_{12} (peptide-based) was measured by fluorescence mode and others by transmission mode. (a) XANES of Pt_{12} subnanoparticles and reference samples. The XANES of Pt_{12} synthesized by the present peptide-based method (white circles) is almost equivalent to that of Pt_{12} synthesized by the previously reported dendrimer-based method (black dots).[1] (b) EXAFS of Pt_{12}/KB and Pt foil measured at room temperature. The black and blue lines of Pt_{12}/KB indicate the EXAFS of dendrimer-based Pt_{12}/KB and the present peptide-based Pt_{12}/KB, respectively. (c) A curve-fitting result (blue: experiment, red: fitting) of dendrimer-based Pt_{12}/KB with a one-shell model considering only a Pt-L (L is C, N or O) shell. (d) A curve-fitting result (blue: experiment, red: fitting) of dendrimer-based Pt_{12}/KB with a two-shell model considering Pt-L (L is C, N or O) and Pt-Pt shells. For (c) and (d), k^3-weighted EXAFS (inset Fig. S) and the Fourier-transformed plots are shown.

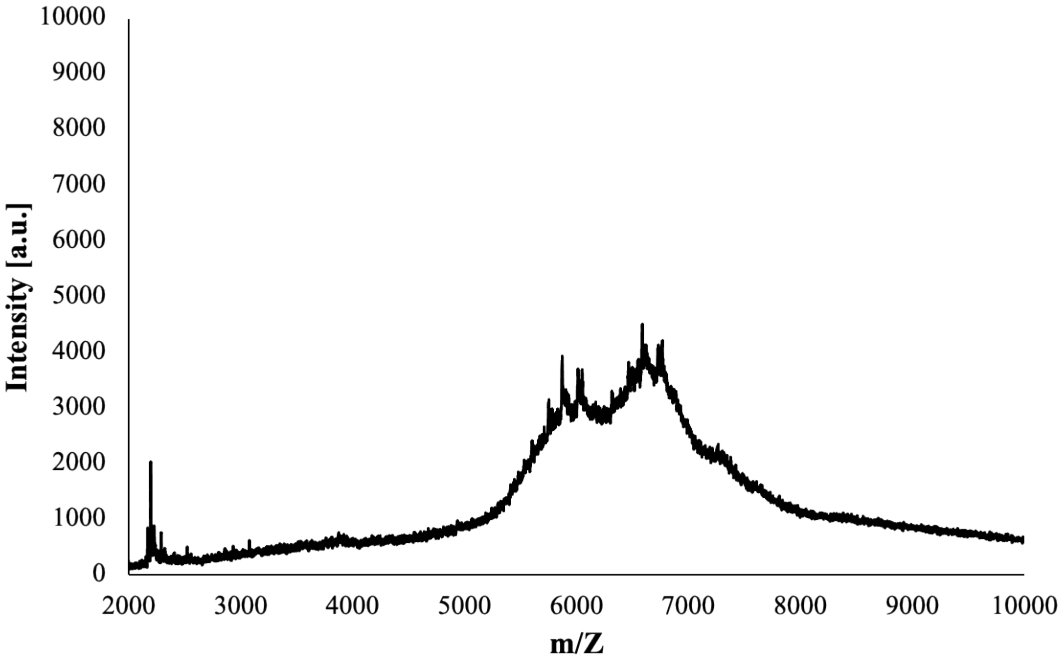

Fig. S10 MALDI-TOF-MS of **Fmoc-(E-Pt)-(K-Fe)-(E-Pt)2-(K-Fe)2-(E-Pt)-(K-Fe)2-(E-Pt)-(K-Fe)-(E-Pt)-RA** synthesized by the SPPS method. Different matrixes, including DCTB (*trans*-2-[3-(4-tert-Butylphenyl)-2-methyl-2-propenylidene]malononitrile), CHCA (α-cyano-4-hydroxycinnamic acid), 4-nitroaniline or dithranol, was used for the matrix.

Table S1 The yields of the catalytic oxidation of toluene the Pt catalysts.

	Product yield (mmol)				
	Benzaldehyde	Benzyl Alcohol	Benzoic Acid	Benzyl Benzoate	Total
Pt_{12}/KB (0.5 wt%)	0.219	0.150	0.473	0.009	0.851
Pt/C (10 wt%)[a)]	0.023	0.010	0.010	0.000	0.043
KB (Blank)[b)]	0.000	0.000	0.000	0.000	0.000

a) Commercially available platinum catalysts on a carbon support for comparison purchased from Sigma Aldrich. The amount of catalyst was set so that the number of moles of platinum was equal to that of Pt_{12}/KB.

b) Ketjenblack support without the platinum catalysts. The amount of support was set to be equal to those of Pt_{12}/KB.
